# PubChem atom environments

**DOI:** 10.1186/s13321-015-0076-4

**Published:** 2015-08-19

**Authors:** Volker D Hähnke, Evan E Bolton, Stephen H Bryant

**Affiliations:** Department of Health and Human Services, National Center for Biotechnology Information, National Library of Medicine, National Institutes of Health, 8600 Rockville Pike, Bethesda, MD 20894 USA

**Keywords:** Molecular graph, PubChem, Fragment, Standardization, SMARTS

## Abstract

**Background:**

Atom environments and fragments find wide-spread use in chemical information and cheminformatics. They are the basis of prediction models, an integral part in similarity searching, and employed in structure search techniques. Most of these methods were developed and evaluated on the relatively small sets of chemical structures available at the time. An analysis of fragment distributions representative of most known chemical structures was published in the 1970s using the Chemical Abstracts Service data system. More recently, advances in automated synthesis of chemicals allow millions of chemicals to be synthesized by a single organization. In addition, open chemical databases are readily available containing tens of millions of chemical structures from a multitude of data sources, including chemical vendors, patents, and the scientific literature, making it possible for scientists to readily access most known chemical structures. With this availability of information, one can now address interesting questions, such as: what chemical fragments are known today? How do these fragments compare to earlier studies? How unique are chemical fragments found in chemical structures?

**Results:**

For our analysis, after hydrogen suppression, atoms were characterized by atomic number, formal charge, implicit hydrogen count, explicit degree (number of neighbors), valence (bond order sum), and aromaticity. Bonds were differentiated as single, double, triple or aromatic bonds. Atom environments were created in a circular manner focused on a central atom with radii from 0 (atom types) up to 3 (representative of ECFP_6 fragments). In total, combining atom types and atom environments that include up to three spheres of nearest neighbors, our investigation identified 28,462,319 unique fragments in the 46 million structures found in the PubChem Compound database as of January 2013. We could identify several factors inflating the number of environments involving transition metals, with many seemingly due to erroneous interpretation of structures from patent data. Compared to fragmentation statistics published 40 years ago, the exponential growth in chemistry is mirrored in a nearly eightfold increase in the number of unique chemical fragments; however, this result is clearly an upper bound estimate as earlier studies employed structure sampling approaches and this study shows that a relatively high rate of atom fragments are found in only a single chemical structure (singletons). In addition, the percentage of singletons grows as the size of the chemical fragment is increased.

**Conclusions:**

The observed growth of the numbers of unique fragments over time suggests that many chemically possible connections of atom types to larger fragments have yet to be explored by chemists. A dramatic drop in the relative rate of increase of atom environments from smaller to larger fragments shows that larger fragments mainly consist of diverse combinations of a limited subset of smaller fragments. This is further supported by the observed concomitant increase of singleton atom environments. Combined, these findings suggest that there is considerable opportunity for chemists to combine known fragments to novel chemical compounds. The comparison of PubChem to an older study of known chemical structures shows noticeable differences. The changes suggest advances in synthetic capabilities of chemists to combine atoms in new patterns. Log–log plots of fragment incidence show small numbers of fragments are found in many structures and that large numbers of fragments are found in very few structures, with nearly half being novel using the methods in this work. The relative decrease in the count of new fragments as a function of size further suggests considerable opportunity for more novel chemicals exists. Lastly, the differences in atom environment diversity between PubChem Substance and Compound showcase the effect of PubChem standardization protocols, but also indicate that a normalization procedure for atom types, functional groups, and tautomeric/resonance forms based on atom environments is possible. The complete sets of atom types and atom environments are supplied as supporting information.

**Graphical abstract:**

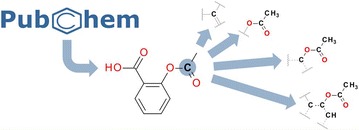

**Electronic supplementary material:**

The online version of this article (doi:10.1186/s13321-015-0076-4) contains supplementary material, which is available to authorized users.

## Background

The de facto standard for the representation of small molecules in chemical information and cheminformatics is the molecular graph, a mathematical construct providing the topological description of a chemical structure as a set of vertices (corresponding to atoms), and edges between those vertices (corresponding to bonds between atoms) [1, 2]. The molecular graph is deeply rooted in valence bond theory, where the structure diagram is (essentially) equivalent to the Lewis structure of a molecule [3, 4]. It helps provide the basis for several related chemical descriptions: systematic names [5–7], line notations [8–22], and connection table-based file formats [23–26]. The valence bond model description of a chemical structure has proven to be incredibly useful to chemists, even though it is simplistic compared to a full quantum mechanical description. Subgraphs, referred to as substructures or molecular fragments, are the key concept in a variety of standard methods for the assessment of chemical similarity [27–34], clustering [35–39], and structure searching [40–42]. For example, fragment-based approaches of atom-centered or variable topological characteristics are used to accelerate chemical structure searches in databases [43–45].

**Fig. 1:**
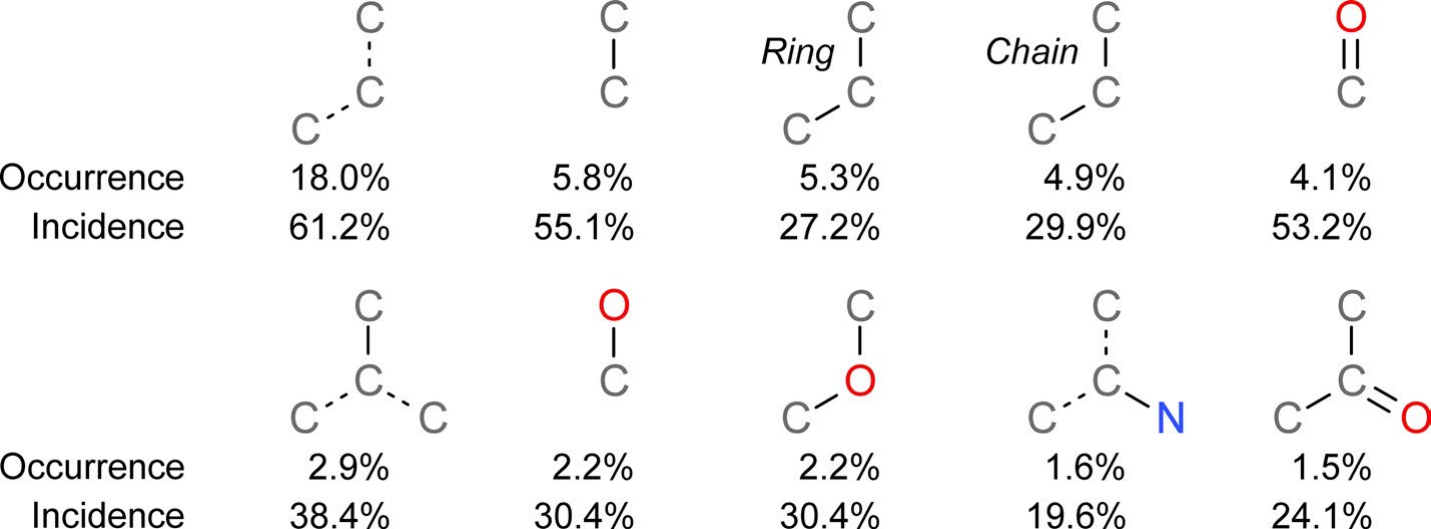
Historic atom environment analysis. Most frequent atom environments as described by Adamson et al. [47]. The term ‘incidence’ refers to the percentage of structures in the survey that contained a particular atom environment, ‘occurrence’ describes the fraction of all generated atom environments that were of the particular type. Incidence was used for ranking. *Dashed line* indicates aromatic bond. Adamson et al. did not provide a definition of aromaticity but distinguished between ring and chain bonds. ‘Ring’ and ‘Chain’ refers to the complete atom environment.

**Fig. 2:**
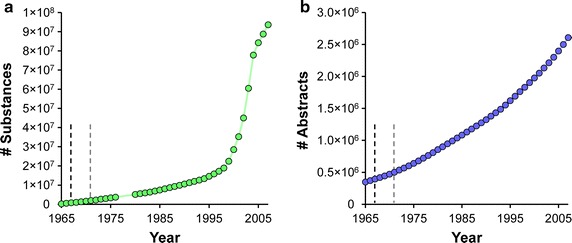
Exponential growth in chemistry. Data reflects registered **a** substances; and **b** abstracts on record at Chemical Abstracts Service at year end [50]. No substances data is available for years 1977–1979. *Black dashed line* indicates time of random sampling of 28,963 structures from the 593,071 individual structures in the Chemical Abstracts Registry System [46]. *Gray dashed line* indicates time of the ‘augmented atom’ analysis by Adamson et al. on this subset [47]. Numbers for abstracts on record include papers, patents and books.

Chemical fragments are interesting in that they can have many uses. More than 40 years ago an atom-centered fragmentation model, referred to as ‘augmented atoms’, was used to analyze 28,799 molecules sampled from the Chemical Abstracts Service Registry System (CASRS) [46, 47]. In that study, 2,331 unique atom environments could be distinguished based on the most discriminative of the employed fragment models, which considered atomic number and seven bond types (single/double/triple bond in a chain or ring, respectively; and aromatic bond in a ring). The resulting top-10 ranked fragments and their incidences (count of structures containing a fragment) and occurrences (count of all cases of a fragment across all structures) from this analysis are reproduced in Fig. 1. One can consider this set of 2,331 augmented atoms a representation of the ability of chemists at that point in time to synthesize and isolate novel chemical substances (with every sampled chemical known to the CASRS at that point in time being some combination of these 2,331 fragments). If the count of scientific publications [48] and known chemical substances [49, 50] illustrated in Fig. 2 are any indication, chemistry as a science has progressed significantly in 40 years. But how have chemists progressed in terms of their ability to synthesize and isolate novel chemistry between then and now? Using this earlier study of CASRS, one could rephrase this question as: what chemical fragments exist in chemical structures today that did not exist then?

In the world of chemical information much has changed since the 1970s. For example, aided by computers and the internet, chemical information data exchange has become increasingly open. There are now chemical data repositories providing access to large quantities of aggregated chemical information without barriers or paywalls. An example of one of these repositories is PubChem.

PubChem is an open archive for chemical substances and their biological activities [51–54]. It consists of three distinct primary databases: Substance, Compound and BioAssay. Substance contains descriptions of chemical substances as provided by hundreds of contributors. BioAssay contains bioactivity information about chemical substances. Compound is derived from entries in Substance via automated protocols that generate a preferred chemical representation and identify equivalent chemicals between PubChem contributors. At the time of initially writing this manuscript (January 2013), PubChem contained more than 100 million substance and 46 million compound records. Given the size and breadth of contributing organizations (including many substance suppliers, patent databases, natural product collections, literature databases, etc.), PubChem might be considered to represent a rather large fraction of all known (small molecule, organic) chemistry.

Using PubChem chemical contents and the earlier analysis method used with CASRS, this study assesses the overall progress by chemists to access novel chemistry over the past 40 years as a function of new, unique chemical fragments. In addition, we present here detailed statistics about atom environments of different sizes in the PubChem Substance (non-standardized structures as provided by contributors) and PubChem Compound (standardized unique structures) databases.

## Results and discussion

### Terminology and approach

Unless stated otherwise, the following approach and definitions were used for the purpose of this study. Incidence refers to the absolute count or percentage of (substance or compound) records that *contain* a particular fragment. Occurrence refers to the absolute count or percentage of *all* fragments across *all* structures (substance or compound) considered. Atom environments are defined as circular atom-centered topological neighborhood fragments of varying ‘radii’ containing all bonds between included atoms and are constructed as detailed in the “Methods”, unless otherwise stated. The ‘radius’ (*r*) of an atom environment is the maximum allowed topological distance between the center atom and any atom in the original structure that is part of the atom environment, and is measured as the number of bonds along the shortest path [55]. The analysis of PubChem Compound and Substance was performed with respect to atom environments from topological radii zero (i.e., atom type) up to three. As a means of comparison, atom environments of *r* = 3 are essentially equivalent to those generated by the popular ECFP_6 type extended connectivity fingerprints [34] or Morgan Fingerprints of *r* = 3 [56]. Atoms are characterized by atomic number, formal charge, implicit hydrogen atom count, explicit degree (number of explicitly connected atoms), and their valence (the sum of implicit hydrogen count and bond orders of incident bonds). Bonds are distinguished as single, double or triple covalent bonds. Both atoms and bonds were further characterized by their participation in aromatic systems. To ensure that these properties are set consistently, pre-processing is performed that converts all explicit hydrogen atoms to implicit hydrogen atom counts (ignoring isotopes and annotated stereo chemistry) and that perceives aromaticity. Atom environment frequencies are specified by incidence, being the absolute number or relative percentage of the 104,669,789 substance or 46,704,121 compound records considered in this study where a molecular fragment is present.

### Known Chemistry (*‘then and now’*) Comparison

To contrast the current state of known chemistry (*‘now’*) with that from a little more than 40 years ago (*‘then’*), we generated atom environments of radius *r* = 1 for all structures in PubChem Compound. To achieve a direct comparison, we used the same atom and bond types as Adamson et al. [47] when analyzing the Crowe et al. data [46]. In these earlier fragmentation studies: atoms were distinguished by atomic number; and bonds were classified as single/double/triple and as chain/ring bonds, respectively, and by an aromatic-ring bond type. For better discrimination between this then to now comparison from other results of our study, this particular size of atom environment generated with these particular atom types and bond types will be referred to as ‘augmented atoms’, as in the 1971 study. Based on this classification scheme, the 1971 study found a total of 2,331 unique augmented atoms in a collection of 28,799 molecules randomly sampled from the CASRS. To ensure comparability, we applied the same pre-filtering steps to structures as performed in the original study: entries with more than 100 atoms were omitted, and only structures containing atoms between 1 and 4 explicit connections were allowed, yielding 46,605,207 allowed and 98,914 rejected compound records from the 46,704,121 compound records in PubChem. In addition, terminal atoms were allowed as center atoms of atom environments. Aromaticity was perceived using the OEChem C++ toolkit [57] aromaticity model OEAroModelMDL, which allows only six-membered rings of carbon and nitrogen to be aromatic, provided they satisfy the ‘Hückel 4n + 2′ rule [58, 59] (i.e., atoms are sp^2^-hybridized). Even though no aromaticity definition was supplied in the 1971 study, due to its simplicity, it is our opinion that this model might be closest to the perception of aromaticity at that time [60].

Using this earlier analysis method, 18,381 unique *r* = 1 augmented atoms were identified in PubChem Compound. (Please note that not adhering to the original study’s pre-filtering steps has virtually no effect on the results with respect to incidences and occurrences; however, the number of augmented atoms increases slightly to 18,503.) The most frequent augmented atom is part of an aromatic system and contains three aromatic carbon atoms and an implicit hydrogen atom (represented as ‘c:c:c’ in SMILES). Compared to an incidence of 61.2% and an occurrence of 18.0% in 1971, today it is present in 83.7% of all structures and accounts for 20.3% of all fragments. This indicates that today a higher fraction of known structures contain aromatic systems. Further evidence can be found by looking at the augmented atom ‘c:c(–C):c’, a ‘branching’ fragment from an aromatic system, ranked sixth most frequent in 1971 is now third most frequent today. Its incidence grew from 38.4 to 66.7%, meaning that this augmented atom is present in almost twice as many structures today. In accordance with these findings, its occurrence increased from 2.9 to 4.6%. Unfortunately, the study by Adamson et al. from 1971 does not contain a complete list of identified augmented atoms or the list of original structures. Consequently, we were unable to duplicate their study and perform a detailed comparison of the results to identify augmented atoms unique to each of the sets. Such a comparison could possibly identify changes in utilized and preferred chemistry with greater specificity. However, the juxtaposition of frequencies of elements encountered in both repositories (for the 1971 analysis, data published by Crowe et al. [46] is used) as presented in Table 1 reveals similarities and differences at the level of element distribution.

**Table 1:** Elemental analysis and comparison

CASRS (1970)			PubChem compound (2013)		
Atomic symbol	Occurrence (%)	Incidence (%)	Atomic symbol	Occurrence (%)	Incidence (%)
C	74.006	99.644	C	74.129	99.922
O	13.519	82.578	N	10.238	91.629
N	7.258	64.165	O	11.288	91.525
F	1.690	10.020	S	1.564	34.171
S	1.302	19.925	Cl	0.933	19.184
Cl	1.208	14.032	F	1.350	18.153
P	0.282	n.s.	Br	0.266	6.468
Br	0.262	n.s.	P	0.060	1.159
Si	0.114	n.s.	Si	0.056	1.058
I	0.077	n.s.	I	0.041	0.968
B	0.063	n.s.	B	0.017	0.335
Sn	0.026	n.s.	Na	0.012	0.211
Se	0.020	n.s.	Y	0.004	0.071
As	0.020	n.s.	Sn	0.003	0.064

Incidence is used for ranking. Occurrence is calculated based on counts of non-hydrogen atoms. Incidence refers to the number of structures in the respective repository that contained at least one atom of the particular element. Frequencies determined in PubChem Compound are compared to a 1970 analysis of the Chemical Abstracts Service Registry System (CASRS) by Crowe et al. [46]. Data not supplied by Crowe et al. is indicated as ‘not specified’ (n.s.). Even though the CASRS data was generated using only 28,963 of 596,367 available compounds, the authors found their data to be nearly identical with that obtained by others from the full set.

Comparing *then* (1970 Crowe et al. study [46]) and *now* (2013 PubChem Compound), carbon is unchanged. It is the most abundant element found in chemical structures, accounting for 74% of all atoms and found in more than 99% of all structures. The story is different for oxygen. It accounts for a decreased percentage of all atoms (13.5% *then* and 11.3% *now*) but, interestingly, the fraction of structures containing oxygen have increased (82.6% *then* and 91.5% *now*). The change is even more dramatic for nitrogen. It accounts for a substantially larger percentage of all atoms (7.3% *then* and 10.2% *now*) and is present in substantially more chemical structures (64.2% *then* and 91.5% *now*). Combined, these three elements (C, N, O) account for nearly all atoms both *then* (94.8%) and *now* (95.7%). Other noteworthy changes include the increased presence in structures the elements sulfur (19.9% *then* and 34.2% *now*) and fluorine (10.0% *then* and 18.1% *now*). The reported incidence of other elements was limited in the 1971 study, preventing a more complete analysis.

**Fig. 3:**
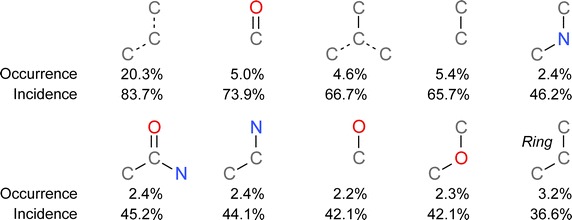
Top-10 most frequent ‘augmented atoms’ in PubChem Compound. PubChem Compound records were fragmented with atom type and bond type definitions identical to those used by Adamson et al. [47]. Incidence was used for ranking, calculated based on 46,605,207 compound records. Dashed lines indicate aromatic bonds as perceived using the aromaticity model OEAroModelMDL in the OpenEye Scientific Software, Inc. OEChem C++ toolkit [57].

Comparison of the top-10 augmented atoms between *then* (Fig. 1) and *now* (Fig. 3) is very interesting. They show oxygen double bonded to carbon (‘O=C’) jumping from a ranking of 5th to 2nd. Three new *now* top-10 augmented atoms (in terms of incidence) appear, all containing nitrogen, including secondary amine (‘C–N–C’, ranked 5th), amide (‘C–C(=O)–N’, ranked 6th), and aliphatic amine (‘C–C–N’, ranked 7th). These replace the *then* augmented atom of aliphatic carbon chain (‘C–C–C’, ranked 4th), aromatic amine (‘c:c(–N):c’, ranked 9th), and aliphatic carbonyl (‘C–C(=O)–C’, ranked 10th). In addition, between *then* and *now* the 6th ranked fragment bumps up to 3rd and the 2nd, 7th, 8th, and 3rd ranked fragments *then* decreased to become the 4th, 8th, 9th, and 10th ranked fragments *now*, respectively.

Beyond the top-10, this comparison shows that since 1970 the number of unique augmented atoms generated has increased by a factor of 7.9. (As stated later, this factor of 7.9 should be considered an upper bound estimation.) This increase in fragment diversity shows that, in the last 40 years, chemists have increased their ability considerably to generate unique combinations of elements and their binding patterns. In addition, and as reflected in the top-10, chemists have become much more adept at working with organic chemicals containing oxygen and nitrogen bonds to the point that many chemicals now contain these. (See also part of the “Atom environment rate of growth” below for discussion on over and under estimation of atom environments in this analysis.) For completeness, the full data of the elemental analysis of Compound is provided as supporting information in Additional file 1: Figure S1 and Table S1. The full list of augmented atoms used in this *then* and *now* comparison with their respective incidence is provided as supporting information in Additional file 2.

### Analysis of PubChem Substance and Compound

Analysis of PubChem chemical substance descriptions (PubChem Substance: 104,669,789 records) and the unique set of chemical structures after PubChem normalization processing (PubChem Compound: 46,704,121 records) was performed. These two collections were examined according to the data preprocessing and analysis approach as described in the “Methods”. Atoms were characterized by atomic number, formal charge, implicit hydrogen count, explicit degree (number of neighbors), valence (bond order sum including implicit hydrogen atom counts), and aromaticity. Bonds were differentiated as single, double, triple, or aromatic bonds. Atom environments were generated for radii *r* = 0 (atom types), *r* = 1, *r* = 2, and *r* = 3, where the topological radius (*r*) is the maximum allowed topological distance between the center atom and any atom in the original structure that is part of the atom environment, as measured by the number of bonds along the shortest path [55].

**Fig. 4:**
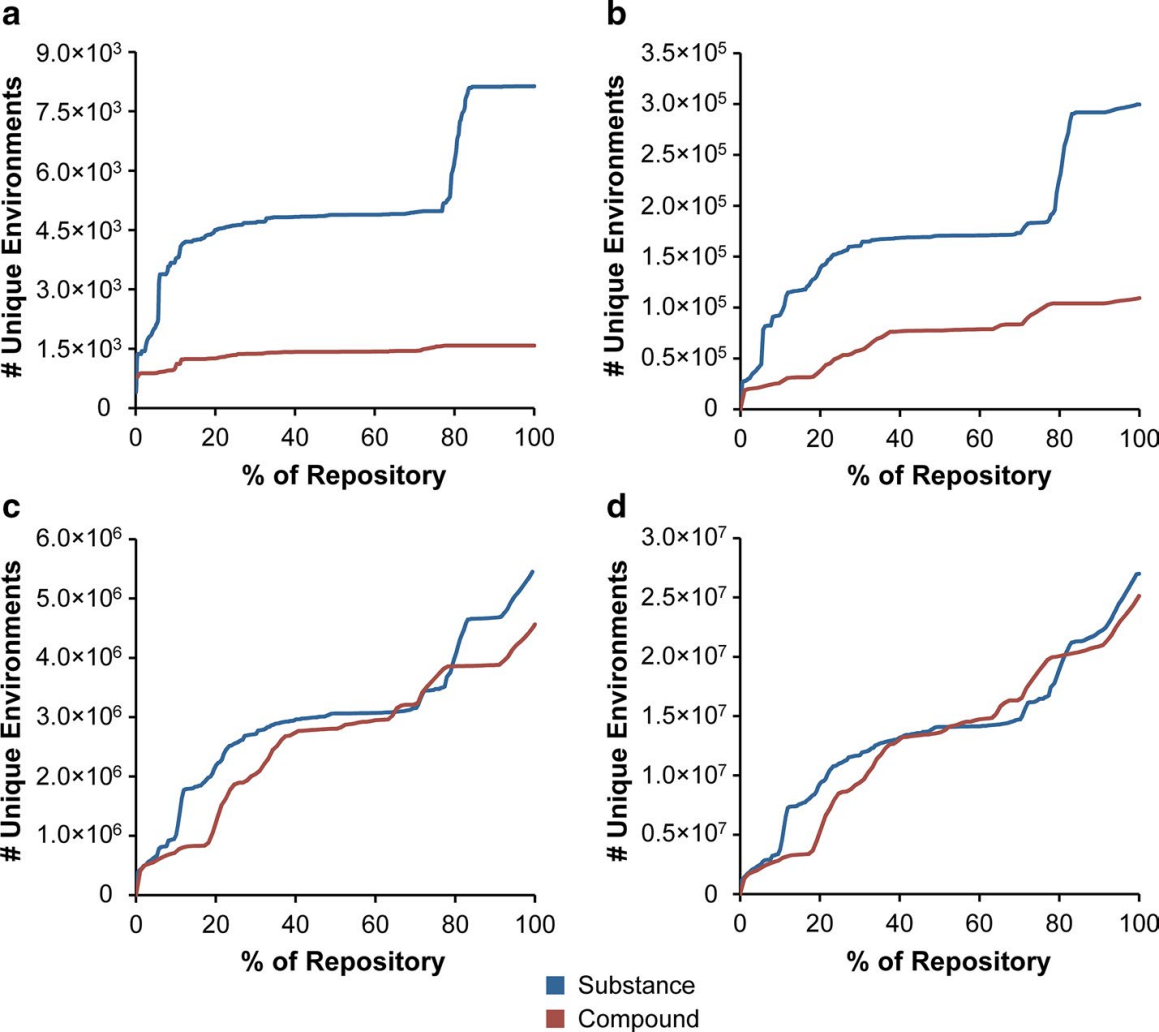
Atom environment statistics. Number of unique atom environments with radius: **a** *r* = 0 (atom types), **b** *r* = 1; **c** *r* = 2; and **d** *r* = 3. Values are per fraction of the respective database, PubChem Substance (104,669,789 SIDs), and PubChem Compound (46,704,121 CIDs). Total fragment counts for *r* = 0,1,2,3 are: 8,135 atom types, 299,609, 5,453,889, and 26,988,962 atom environments, respectively, for Substance; and 1,583 atom types, 109,306, 4,559,587, and 25,115,177 atom environments, respectively, for Compound.

PubChem Substance contained 8,135 (*r* = 0), 299,609 (*r* = 1), 5,453,889 (*r* = 2), and 26,988,962 (*r* = 3) unique atom environments, respectively. PubChem Compound contained 1,583 (*r* = 0), 109,306 (*r* = 1), 4,559,587 (*r* = 2), and 25,115,177 (*r* = 3) unique atom environments, respectively. In Fig. 4, the growth of the number of unique atom types and atom environments in Substance and Compound with increasing substance identifier (SID) and compound identifier (CID) are shown. One cannot read too much into Fig. 4, as the plot of increasing identifier indicates only a historic growth of atom environments with respect to when new/unique records were added to PubChem. For example, new contributors may give “new” structures to PubChem that were known already for some time. When examining Fig. 4, it may seem counterintuitive that for radii *r* = 2 (Fig. 4c) and *r* = 3 (Fig. 4d) a particular fraction of Compound has more unique atom environments than Substance; however, there is no direct correlation between the percentage of Substance and Compound, per se, due to the duplicity and order of chemical structures in Substance. Compound is the unique content of Substance (after PubChem normalization processing) and Substance contains more than twice the number of records as Compound (104 million SIDs vs. 46 million CIDs, at the time of this study). Therefore, there should be no assumption that there is a direct correlation between the two database percentages.

### Atom type (*r* = 0 atom environment) statistics

**Fig. 5:**
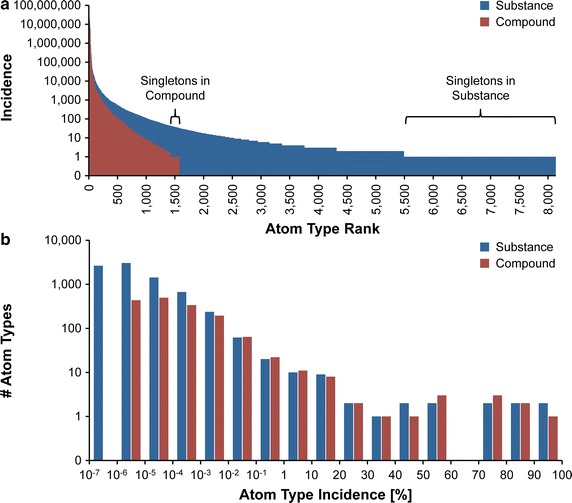
Atom type (r = 0) statistics. **a** Rank/frequency plot resulting from ranking atom types by their incidence (*y*-axis logarithmic). **b** Histogram of incidence percentages; minima inclusive, maxima exclusive; range from 10^−7^ to 10% logarithmic and linear 10–100% for clarity.

**Fig. 6:**
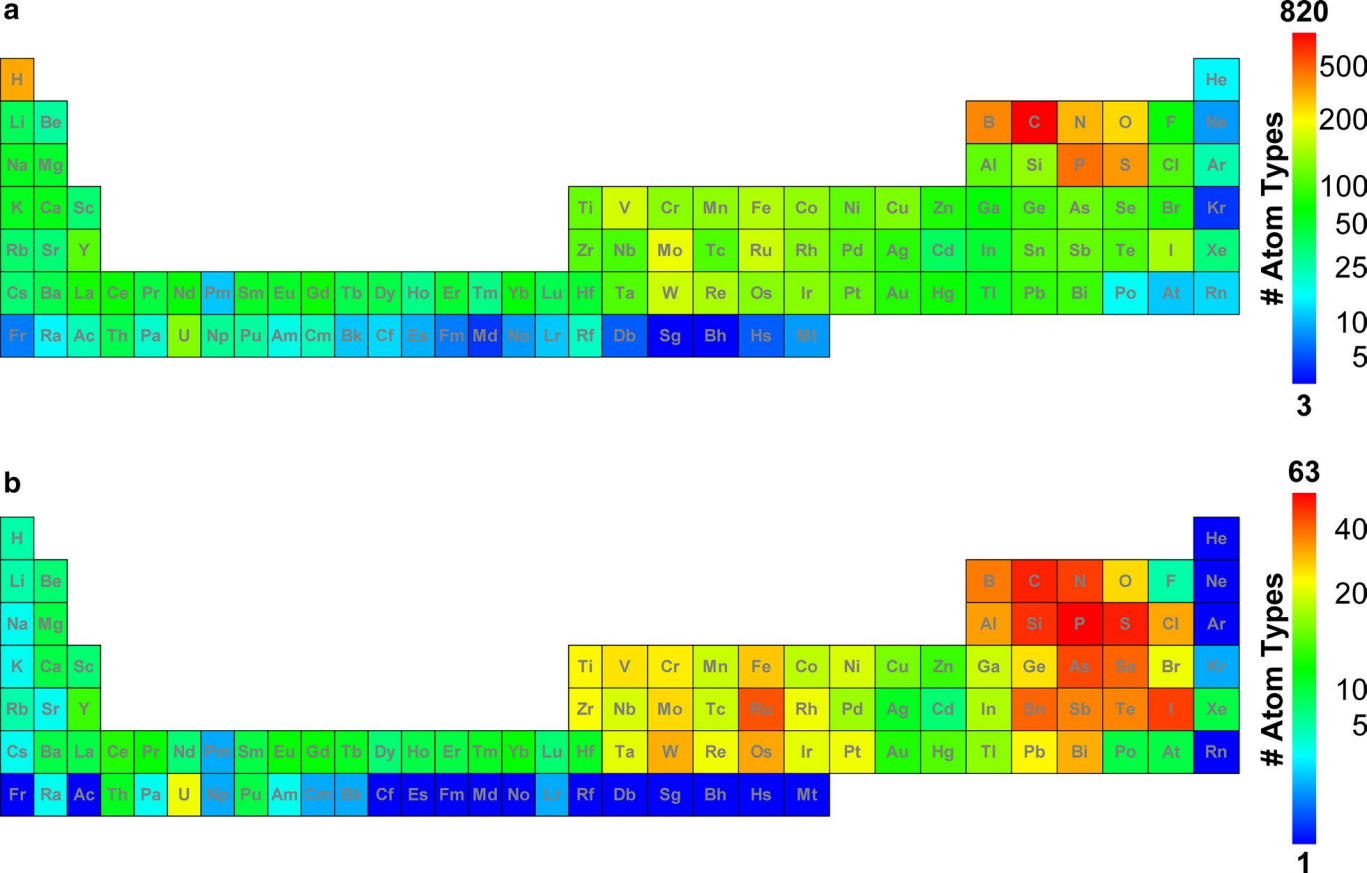
Atom type (r = 0) counts per element. Number of atom types per element. **a** Substance; **b** Compound. Color coding normalized to the respective highest/lowest atom type count for clarity. Logarithmic-based color scale is used for better differentiation between low counts.

PubChem Substance and Compound contain atom types (*r* = 0) of all elements from atomic number 1 (hydrogen) to 109 (meitnerium). In total, 8,135 different atom configurations occur in Substance, with 2,644 (32.5%) being singletons (i.e., occurring in only one substance record). These respective numbers are significantly lower for Compound, which contains 1,583 different atom types, 167 (10.6%) of which are singletons. Rank/frequency plots for atom types in Substance and Compound are shown in Fig. 5. Their dispersal among elements is found in Fig. 6. In total, the largest number of different atom types in the Substance database is based on carbon (820 atom types, 10.1%), while in the Compound database, the largest number is based on phosphorous (63 atom types, 4.0%).

**Fig. 7:**
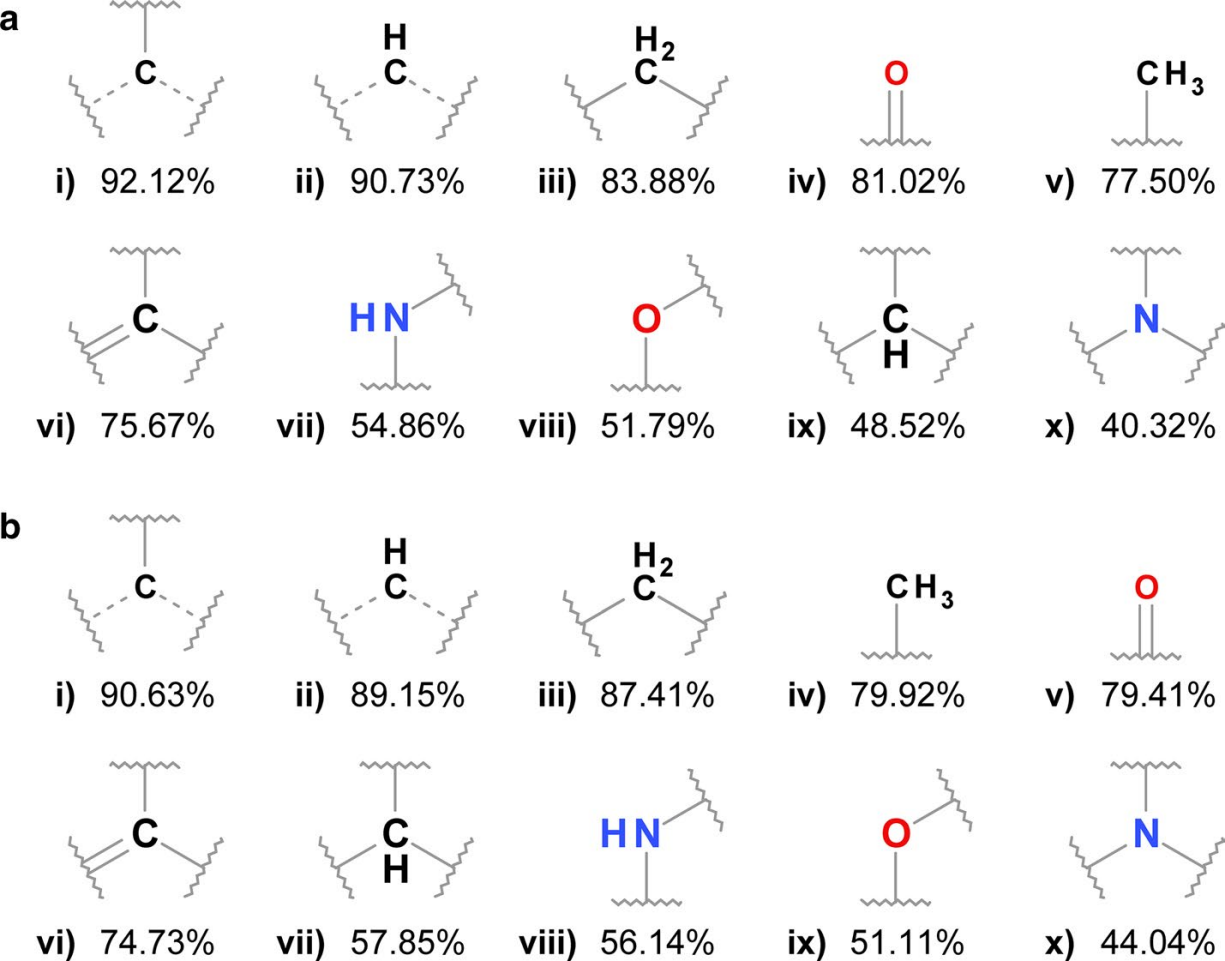
Top-10 most frequent atom types (r = 0) in PubChem. Percentages indicate incidence of the atom type in the respective database: **a** Substance; and **b** Compound. *Light gray bonds* for clarification of connectivity and valence. *Dashed lines* indicate aromatic bonds as perceived using the aromaticity model OEAroModelOpenEye in the OpenEye Scientific Software, Inc. OEChem C++ toolkit [57].

The top-10 most frequent atom types (*r* = 0) in Substance and Compound are shown in Fig. 7. Similar to the earlier ‘*then* and *now’* comparison, all of these top-10 atom types are based on carbon, nitrogen or oxygen. In addition, these atom types are identical between Substance and Compound databases but ranked in a slightly different order, in part, due to the duplicity of the chemical structures in Substance records. Figure 7 also shows that a very small minority of atom types (eight in Substance and nine in Compound) occur in more than 50% of all records in the respective database. The two most frequent atom types contain carbon in an aromatic system, one being additionally bonded to a non-hydrogen atom (Substance 92.1%; Compound 90.6%) and the other to a hydrogen atom (Substance: 90.7%; Compound 89.2%). If aromaticity is ignored, the Substance atom types (i) and (vi) and Compound atom types (i) and (vi) would be identical.

**Fig. 8:**
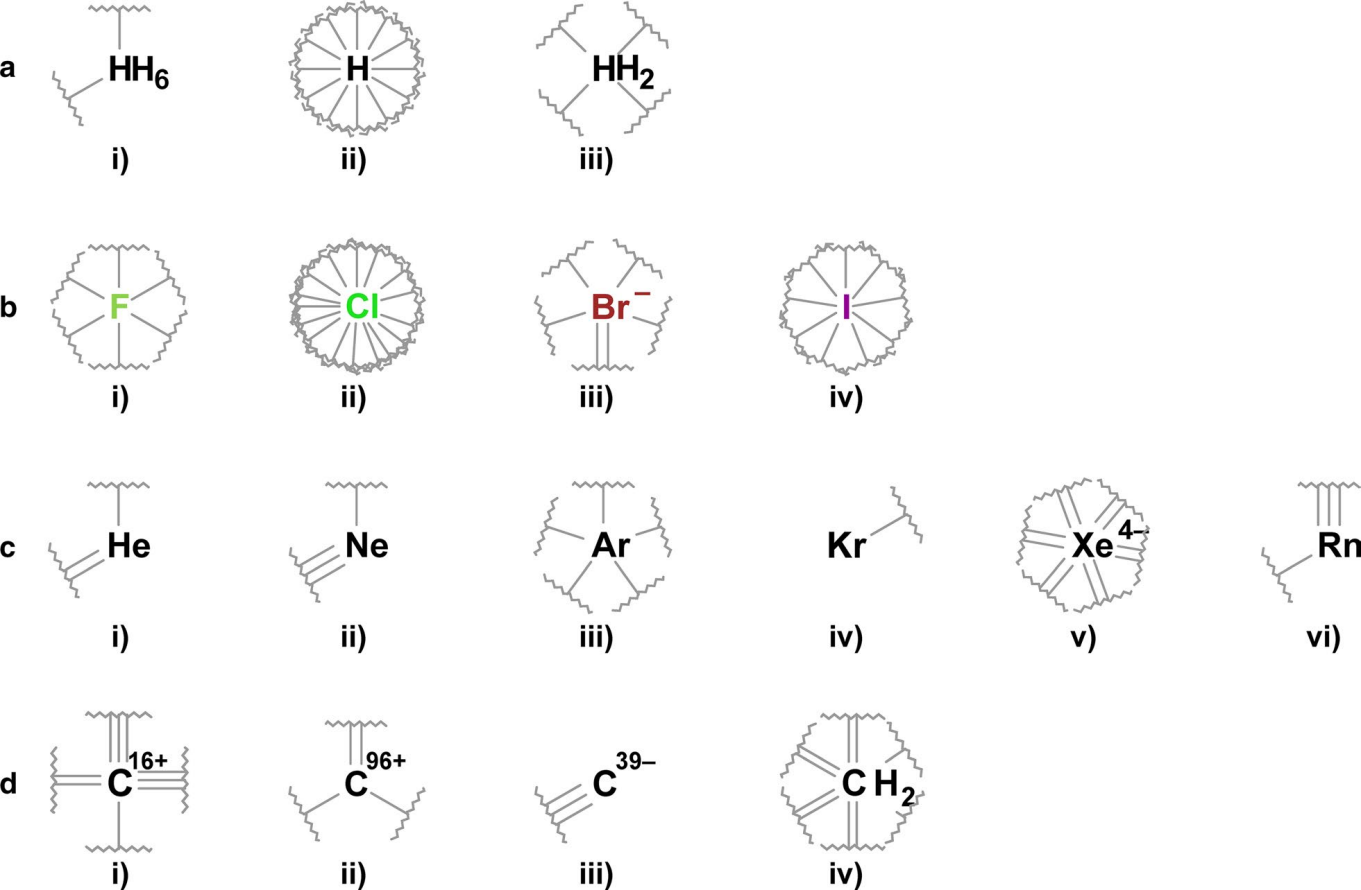
Examples for atom types (r = 0) unique to PubChem Substance. **a** Hydrogen singletons; *i* uncharged, octavalent and di-coordinated hydrogen with six implicit hydrogen atoms (SID 136120614); *ii* uncharged, dodeca-valent and dodeca-coordinated hydrogen with no implicit hydrogen atoms (SID 138472568); *iii* uncharged, hexa-valent and tetra-coordinated hydrogen with two implicit hydrogen atoms (SID 137447009). **b** Halogen singletons; *i* uncharged, hexa-valent and hexa-coordinated fluorine with no implicit hydrogen atoms (SID 35048788), *ii* uncharged, hexadeca-valent and hexadeca-coordinated chlorine with no implicit hydrogen atoms (SID 7802012), *iii* hexa-valent and penta-coordinated bromine with no implicit hydrogen atoms and a charge of −1 (SID 16021530); *iv* uncharged, ennea-valent and ennea-coordinated iodine without implicit hydrogen atoms (SID 142144341). **c** Noble gas singletons; *i* uncharged, tri-valent and di-coordinated helium with no implicit hydrogen atoms (SID 141317149), *ii* uncharged, tetra-valent and di-coordinated neon with no implicit hydrogen atoms (SID 140017906), *iii* uncharged, penta-valent and penta-coordinated argon with no implicit hydrogen atoms (SID 138071622), *iv* uncharged, mono-valent and hen-coordinated krypton with no implicit hydrogen atoms (SID 140411176), *v* dodeca-valent and hexa-coordinated xenon with no implicit hydrogen atoms and a charge of −4 (SID 135041562), *vi* uncharged, tetra-valent and di-coordinated radon with no implicit hydrogen atoms (SID 140679519). **d** Carbon singletons, *i* ennea-valent and tetra-coordinated carbon with no implicit hydrogen atoms and a charge of +16 (SID 142885273), *ii* tetra-valent and tri-coordinated carbon with no implicit hydrogen atoms and a charge of +96 (SID 142278854), *iii* tri-valent and hen-coordinated carbon with no implicit hydrogen atoms and a charge of −39 (SID 139340022), *iv* dodeca-valent and hexa-coordinated carbon with two implicit hydrogen atoms (SID 142657677). Bonding scenarios are replicated as encountered in the respective deposited structures.

The 8,135 atom types reflect the heterogeneity of chemical representations in Substance but also the bizarre. Hydrogen is present in no less than 324 configurations of which 166 (51.2%) are singletons; exemplary atom types unique to Substance are shown in Fig. 8a. PubChem standardization protocols reject 320 of the 324 encountered hydrogen configurations. As such, only four different hydrogen-only atom types exist in Compound: monohydrogen radical, elemental hydrogen in H_2_, hydride ion (H^−^), and hydron ion (H^+^). It may be noteworthy that these hydrogen species are present in the Compound database as explicit atoms despite (explicit to implicit) hydrogen suppression procedures.

In the case of halogen atom types found in Substance, there are 51 for fluorine [16 (31.4%) singletons], 74 for chlorine [16 (21.6%) singletons], 123 for bromine [56 (45.5%) singletons] and 57 for iodine [17 (29.8%) singletons]. Exemplary atom types unique to Substance are shown in Fig. 8b. In Compound, the number of different halogen atom types is substantially lower, with 4 fluorine atom types [0 singletons], 32 chlorine atom types [7 (21.9%) singletons], 49 bromine atom types [12 (24.5%) singletons] and 21 iodine atom types [5 (23.8%) singletons].

Even though noble gases initially were thought to be chemically inert, today, a few noble gas compounds are known [61–67]. Nonetheless, the 206 noble gas atom types identified in Substance seems irrationally high. Exemplars of atom types per noble gas unique to Substance are shown in Fig. 8c.

The 2,644 singleton atom types in Substance can highlight peculiar configurations. For instance, in the case of carbon, there are 351 singletons (13.3 % of all singleton cases), with formal charges ranging from −99 to +291 and valences ranging from 0 to 254 (examples shown in Fig. 8d). For other elements, the situation is similar, as illustrated for organic elements (H, C, N, O, F, P, S, Cl, Br, or I) in Table 2. In total, only 71 of 2,644 (2.7 %) singleton atom types pass the PubChem “allowed” valence list.

**Table 2:** Properties of organic atom type singletons

Atomic number	Atomic symbol	Number singletons	Charge		Valence	
			Minimum	Maximum	Minimum	Maximum
1	H	166	−99	126	0	91
6	C	351	−99	291	0	254
7	N	111	−67	7	0	36
8	O	82	−94	123	0	50
9	F	16	−2	0	1	137
15	P	231	−99	55	0	233
16	S	169	−99	127	0	234
17	Cl	16	−2	5	0	64
35	Br	17	−3	1	0	8
53	I	56	−5	7	0	171

Organic elements are used as examples.

**Fig. 9:**
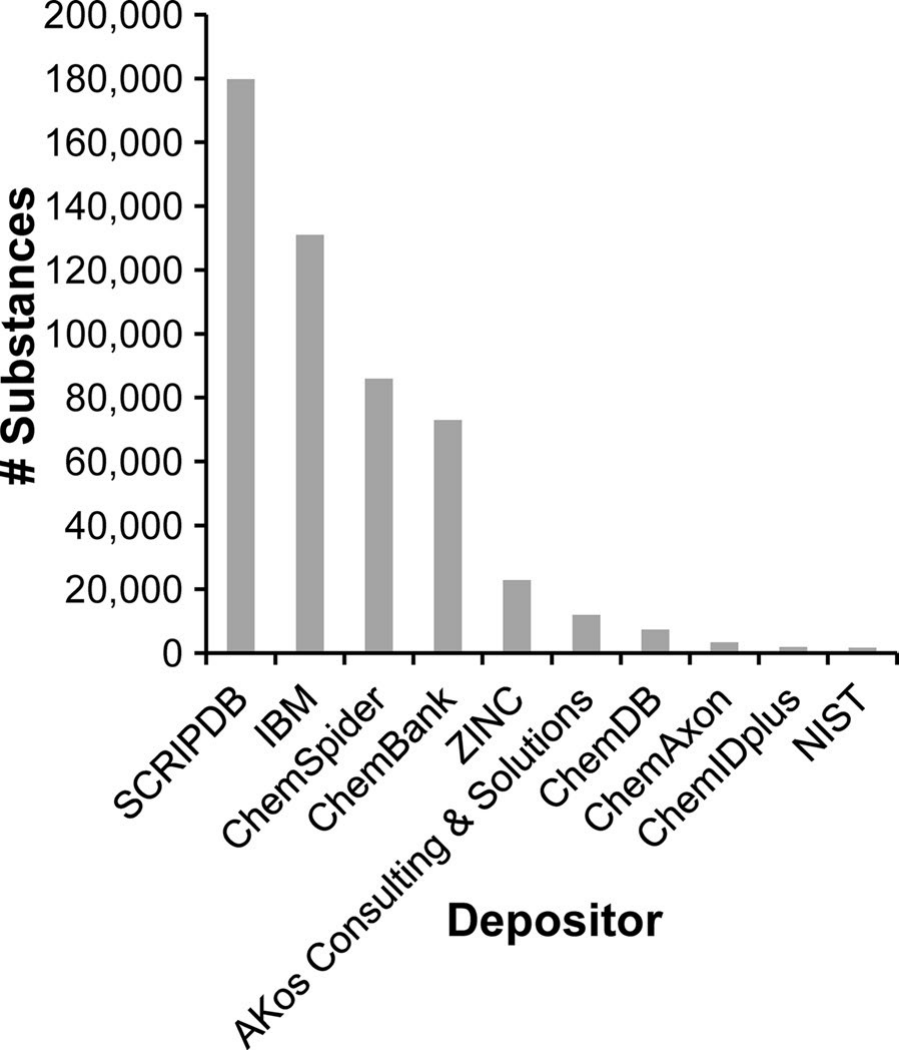
Depositor statistics of invalid atom types (r = 0) in PubChem Substance. Top-10 depositors of substances containing atom types that do not pass the PubChem valence list. Investigated atom types were limited to the 2,727 organic (H, C, N, O, F, P, S, Cl, Br, I) atom types that are connected only to other organic atoms, in order to purposely exclude ‘invalid’ atom types that could result from organic/metal interactions being represented as covalent bonds.

The large number of strange atom types specific to Substance sparked an interest in their origin. Unusual connectivity patterns (and, therefore, valences) are commonly found in depictions between metals and organic elements, where non-covalent interactions are represented using covalent bonds in accordance with the structure representation rules of a given organization. If one ignores such chemistry and considers only the atom types of organic atoms with bonds only to other organic atoms, the total count of 8,135 Substance atom types collapses down to 2,727 (33.5 %). Of these 2,727 Substance “organic-only” atom types, 2,422 (88.8 %) are considered “invalid” by PubChem and 1,144 (47.2 %) of these “invalid organic-only” atom types occur in only a single substance (i.e., are exceedingly rare). In total, 534,968 substances from 119 depositors have one or more of these “invalid organic-only” atom types. The top-10 contributors of these are shown in Fig. 9. Please note that the presence of these “invalid organic-only” atom types does not necessarily indicate a lapse of quality of these resources. It may, in some cases, suggest that PubChem prefers a different chemistry representation than that provided, as “invalid organic-only” includes atom types such as the pervasive pentavalent nitrogen (e.g., as found in the “*N(=O)=O” nitro group representation) commonly found in chemical databases.

**Fig. 10:**
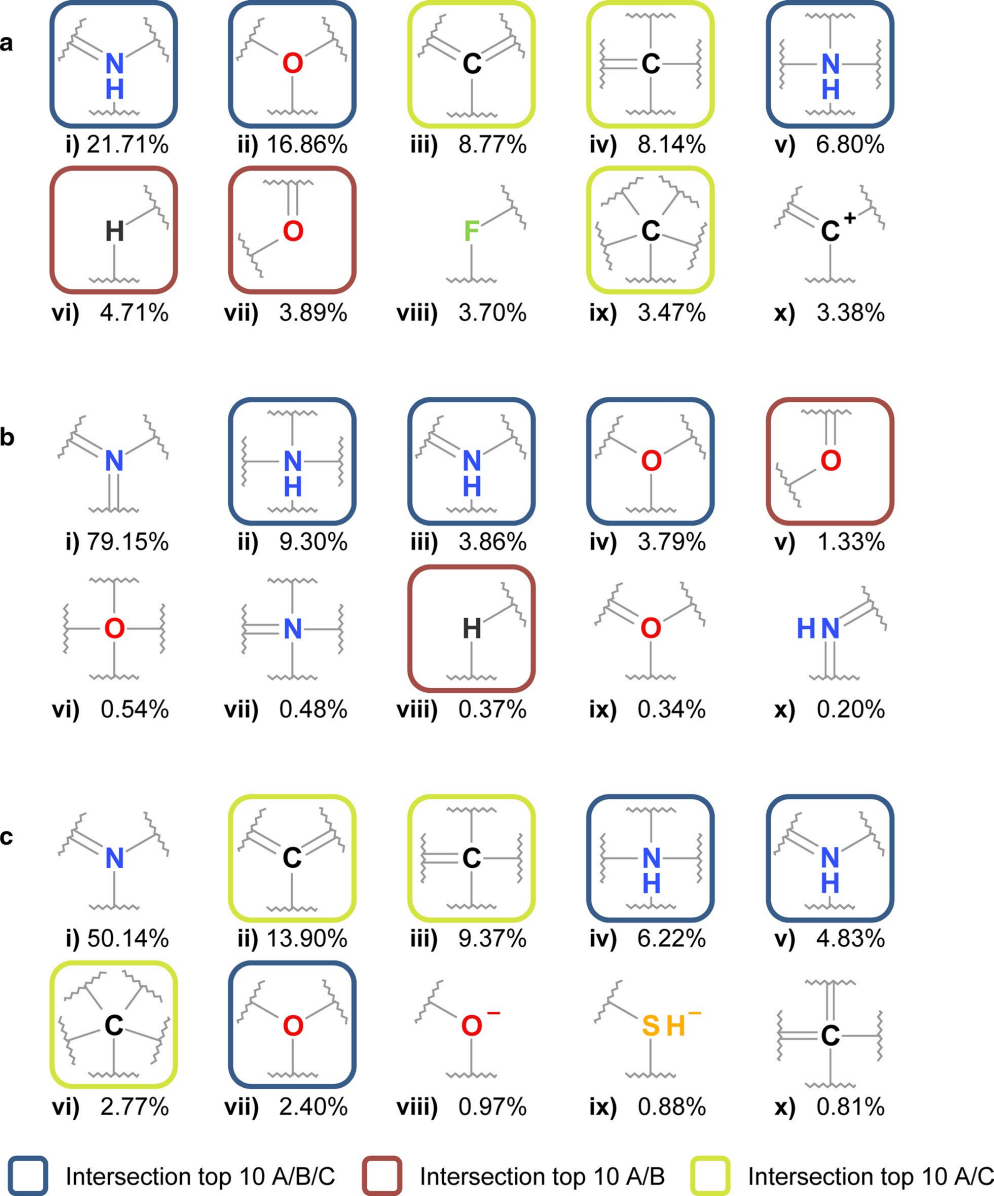
Top-10 most frequent invalid atom types (r = 0) from select data sources. Shown are the top-10 most frequent atom types that fail the PubChem valence list for the top-3 depositors of substances containing failing atom types: **a** SCRIPDB,** b** IBM, and **c** ChemSpider. Atom types are ranked by their relative frequency in substances deposited by the respective contributor that have at least one atom that does not pass the valence list. Atom type incidences were calculated for each depositor separately.

**Fig. 11:**
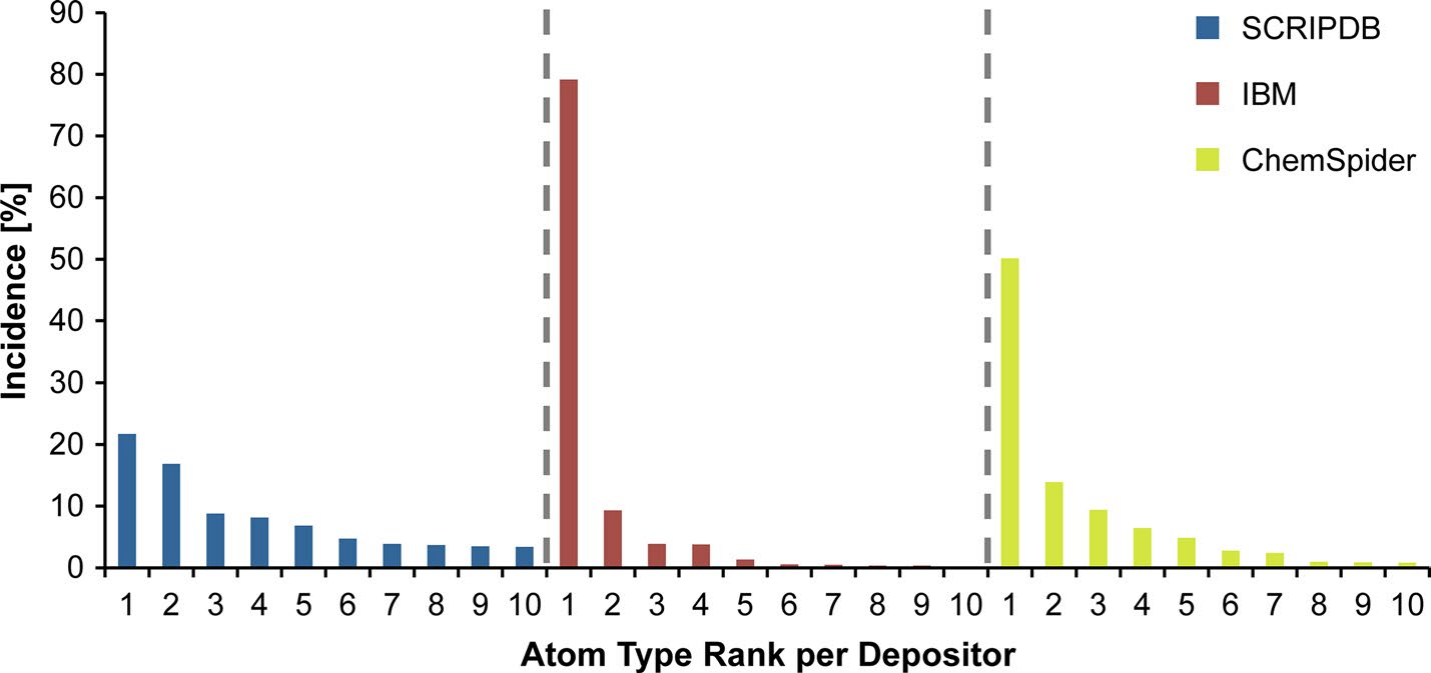
Statistics of invalid atom types (*r* = 0) from select data sources. For the top-3 depositors of substances containing invalid ‘organic-only’ atom types, the incidence of the respective top-10 most frequent invalid atom types is shown. Incidence is calculated for each contributor separately based on the total number of substances containing such atom types. For the corresponding atom types, see Fig. 10.

Nearly 75% of the +500 thousand substances with “invalid organic-only” atom types are from three PubChem contributors: SCRIPDB [179,784 (33.6%)] [68, 69], IBM [131,014 (24.5%)] [70], and ChemSpider [85,894 (16.1%)] [71]. Examples from SCRIPDB are depicted in Figs. 10a (i–x), 8a (ii–iii), b (iv), c (i–iv), (vi), and d (i–iv). Examples from IBM can be seen in Figs. 10b (i–x) and 8a (i). Examples from ChemSpider are found in Figs. 10c (i–x) and 8b (i). For SCRIPDB, IBM and ChemSpider, the relative incidence of the ten most frequent invalid organic-only atom types are presented in Fig. 11 relative to the respective (i–x) atom types depicted in Fig. 10.

In total, 2,181 different “organic-only” atom types encountered in substances deposited by SCRIPDB do not pass the PubChem valence list. SCRIPDB provides chemical structures found in the complex work units (e.g., Figures) of USPTO (United States Patent Office) patent documents. The most frequent one, found in 21.7% of the 179,784 substances with “invalid organic-only” atom types (referred to henceforth as “invalid substances”), is uncharged, tetra-coordinated, tetra-valent nitrogen that gets an added implicit hydrogen atom during pre-processing. This configuration is not allowed in the PubChem valence list, but is salvageable by standardization protocols with a simple fix: the implicit hydrogen is removed during structure standardization, and the nitrogen atom gets assigned a positive charge. The next most frequent atom types are tri-coordinated and tri-valent oxygen (16.9% of invalid substances), tri-coordinated and penta-valent carbon (8.8% of invalid substances), and tetra-coordinated and penta-valent carbon (8.1% of invalid substances). These atom types lead to the rejection of a substance during standardization. The case of tetra-coordinated, tetra-valent and uncharged nitrogen (6.8% of invalid substances) is analogous to the most frequent atom type. Di-coordinated and di-valent hydrogen cases (4.7% of invalid substances) are not salvageable by structure standardization. The same goes for di-coordinated and tri-valent oxygen (3.9% of invalid substances) and di-coordinated and di-valent fluorine (3.7% of invalid substances). Penta-coordinated and penta-valent carbon (3.5% of invalid substances) causes the respective substances also to be rejected. Tri-coordinated, tetra-valent carbon with a positive charge (3.4% of invalid substances) does not exist in the PubChem valence list and is not salvaged by standardization. A total of 1,138 of the 2,181 (52.2%) rejected atom type cases identified in substances provided by SCRIPDB occur in only a single substance record.

There are 116 “invalid organic-only” atom types found in 131,014 substances provided by IBM. The chemical structures from IBM are pulled from patent and biomedical literature documents. Contrary to SCRIPDB, the number of affected substances is dominated by a single atom type: uncharged, tri-coordinated and penta-valent nitrogen, which is present in 79.1% of the affected substances. Inspection of the substances provided to PubChem revealed that this is due to the configuration of nitro groups as “*N(=O)(=O)”, a common representation approach but deemed invalid by PubChem which favors the “*[N+](=O)–[O–]” representation. This is remedied by PubChem standardization, where the configuration of the nitro group with penta-valent nitrogen is modified to be the PubChem-preferred representation. Five of the top-10 ranked “invalid organic-only” atom types are identical with those identified for SCRIPDB in Fig. 10. Substances containing tetra-coordinated and tetra-valent oxygen (0.5% of 131,014 substances) get rejected by standardization. Tetra-coordinated and penta-valent nitrogen is analogous to the most frequent atom type for IBM that is rejected by the PubChem valence list. During structure standardization, this can be resolved if the respective atom is double-bonded to an oxygen atom by modifying this atom type to a charge-separated representation of the double bond to form a tetra-coordinated and tetra-valent nitrogen that carries a positive charge. Tri-coordinated and tetra-valent oxygen (0.3% of 131,014 substances) is rejected by the PubChem valence list. Di-coordinated and tetra-valent nitrogen gets assigned an implicit hydrogen count of +1 during pre-processing (0.2% of 131,014 substances) that later is replaced by a positive charge during standardization. In total, 40 of the 116 (34.5%) invalid atom types identified in substances deposited by IBM occur in only a single substance.

The 85,894 substances with “invalid organic-only” atom types provided by ChemSpider contain at least one of 282 offending atom types. The most frequent one, tri-coordinated tetra-valent nitrogen that is uncharged and has no implicit hydrogen atoms (50.1% of 85 894 substances), is an annotated nitrogen radical. It occurs 8,425 times in the context of nitro groups being represented as “*[N](=O)(–[O^−^])”, and 100 times in other contexts. This representation was previously not handled by the PubChem structure standardization protocols, and consequently, affected substances were rejected. The top-10 ranked invalid atom types identified in substances deposited by ChemSpider share three types with the top-10 ranked atom types from SCRIPDB and IBM, and three other atom types with the top-10 ranked atom types just from IBM. As a complementary invalid atom type, di-coordinated and di-valent oxygen carrying a negative charge occurs in 0.97% of 85,894 substances. These respective cases cannot be salvaged, as it is not clear whether connectivity information or formal charge should have precedence such that this atom type can be modified in order to pass the PubChem valence list. The hydrogen atom in tri-coordinated and tri-valent negatively charged sulfur is actually added during pre-processing, it is deposited as di-coordinated, di-valent and carrying a negative charge. Both configurations are neither in the PubChem valence list nor treated by the standardization protocols and are rejected during standardization. A consequence of the selection criteria for investigated atom types is that all bonds in tetra-coordinated hexa-valent carbon (0.81% of 85,894 substances) are considered to be covalent and this atom type does not pass the valence check during structure standardization. Of the invalid atom types encountered in substances deposited by ChemSpider, 81 of 282 (28.7%) invalid atom types occur in only one substance.

These top-10 cases of “invalid organic-only” atom types help to highlight several things. Firstly, Fig. 10 suggests that simple examination of atom types in a given molecule collection can be helpful to identify molecules that may be considered invalid as depicted. Secondly, as indicated by highlighted overlap in Fig. 10, it demonstrates that different organizations can share or differ in preference for particular “invalid” molecule representations, with each organization potentially providing previous unimagined atom configurations that may or may not be salvageable. Thirdly, some of these atom environments that are technically “invalid” from the PubChem perspective can be readily fixed/normalized to the PubChem preferred atom environment. For example, if one removes an implicit hydrogen atom and adds a positive charge, it would make the nitrogen atom in the Fig. 10a (i, v), b (ii, iii, x), and c (iv, v) cases “valid”. Indeed, PubChem standardization protocols can “correct” the nitrogen atom type as highlighted in these cases, however, it is worth mentioning that such fixes consider the larger atom environment (*r* > 0) and often modify several atom types and respective bonding patterns between them, as opposed to a systemic fix considering only a single atom type (*r* = 0). (Fixes at the *r* = 0 atom environment level should be considered to be ill advised as they may complicate or prevent correction of a larger functional group representation.) Lastly, as suggested in Fig. 11, differences in opinion about preferred representation may affect a large number of structures, but the majority of those differ in only a small number of atom types (in this case, ten or less). The examination of configuration histograms per element between repositories can help to identify such cases and also suggest ways to improve consistency within a given chemical collection.

**Fig. 12:**
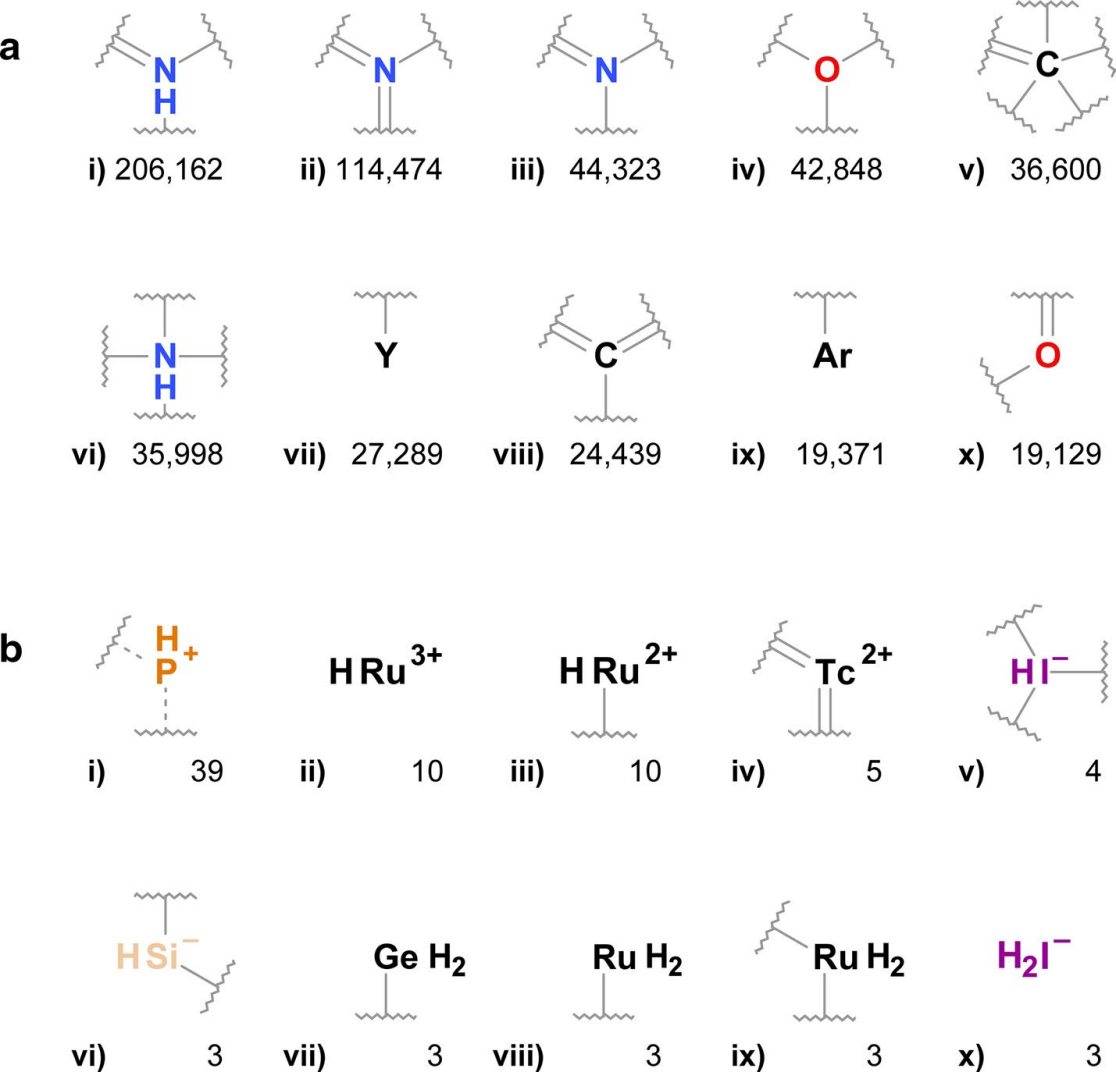
Top-10 most frequent atom types (*r* = 0) unique to Substance and Compound, respectively. Atom types are ranked by their incidence in the respective database: **a** Substance, and **b** Compound. Incidence provided in absolute numbers due to very low corresponding percentages. Please note that **b** (*i*) is aromatic phosphorus and **b** (*vii*) is annotated as a monoradical.

In a more general view across PubChem and without restrictions of considered atom types, a total of 6,583 atom types [of which 2 591 (39.4 %) are singletons] are unique to Substance and do not occur in Compound. The overall top-10 most frequent cases are presented in Fig. 12a. The reasons why they are not found in Compound are similar. In the case of types (i), (iv), (v), (vi), (viii) and (x), one of the covalent single bonds can represent a non-covalent interaction that is converted to a non-standard bond type during PubChem standardization. In addition to that, if all bonds implied in types (i) and (vi) are covalent, the hydrogen atom is removed and a positive charge is placed on the nitrogen atom. Type (ii) frequently occurs in nitro groups with two covalent double bonds to nitrogen. In that case, one of the double bonds is modified to be charge separated. In other cases, analogous to types (v), (viii) and (x), if all bonds are considered to be covalent, the corresponding substance is rejected during standardization. Type (iii) represents a nitrogen radical that is not in the PubChem “allowed” valence list. In the case of type (vii), the covalent single bond to yttrium is converted to a non-standard ‘complex’ bond. Mono-connected mono-valent argon is not in the PubChem allowed valence list, and no standardization rule for this case exists. Consequently, substances containing type (ix) are rejected during standardization.

There are only 31 atom types in Compound that are not found in Substance. The top-10 most frequent examples are shown in Fig. 12b. The first case is a result of the phosphorous atom being part of a non-covalent interaction (complex bond), where the charge is set during structure standardization. Analogously, the configurations found in the examples ranked second, third, fourth, sixth, seventh, eighth and ninth occur when trying to find adequate representations for complex bonds the respective atoms are incident to. The full list of atom types with their respective incidence is provided for Substance and Compound as supporting information in Additional file 3.

### Atom environment (*r* = 1) statistics

**Fig. 13:**
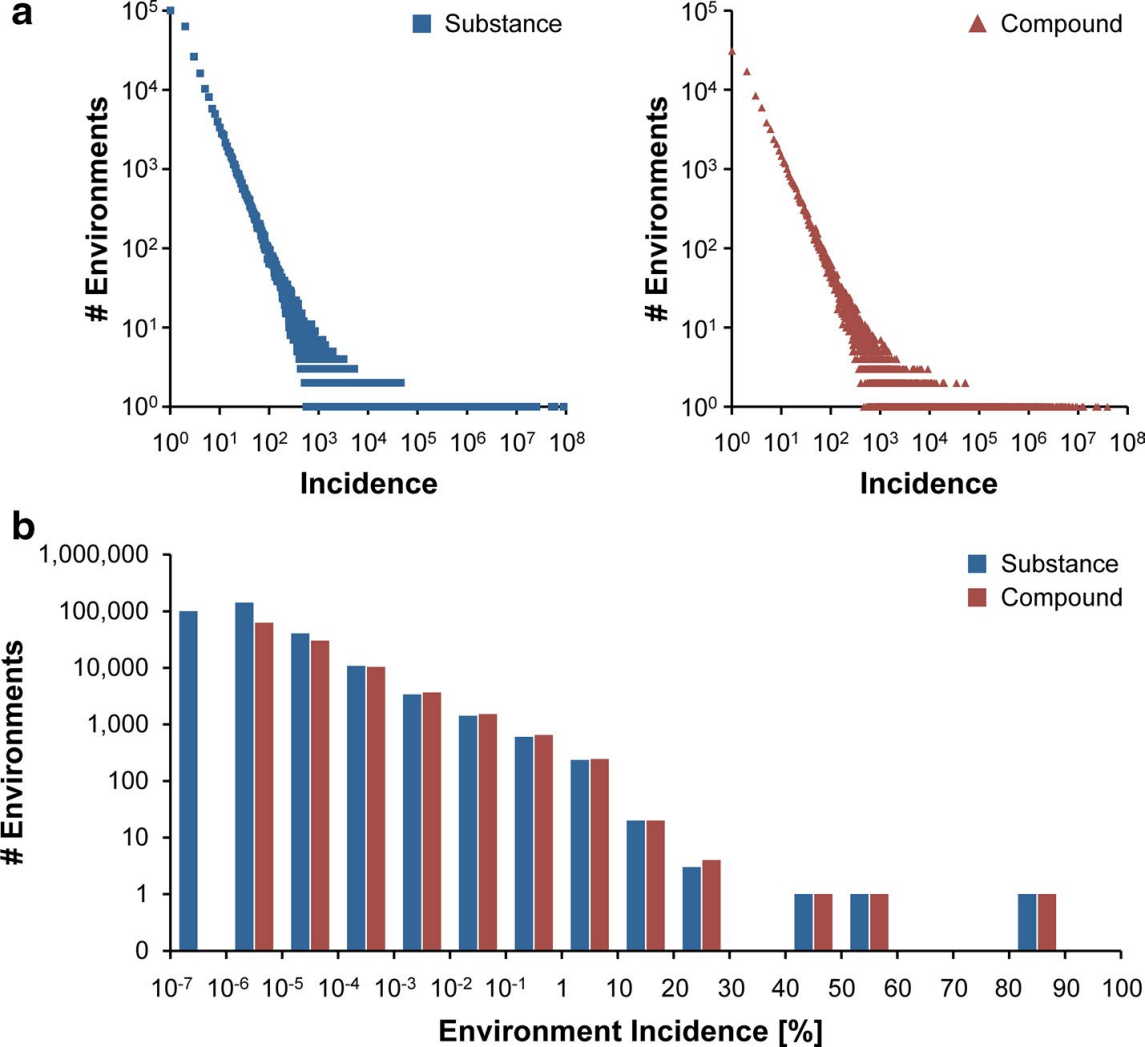
Atom environment statistics radius *r* = 1. **a** Distribution of atom environment incidences (log–log scale plots); **b** Histogram of incidence percentages; minima inclusive, maxima exclusive; range from 10^−7^ to 10% logarithmic and linear 10–100% for clarity.

**Fig. 14:**
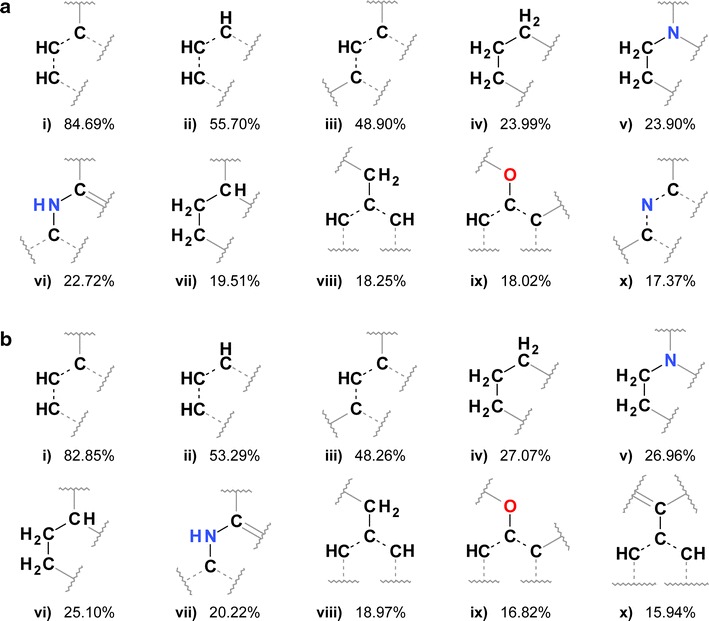
Top-10 most frequent atom environments with radius *r* = 1 in PubChem. *Percentages* indicate incidence of the atom environment in the respective database: **a** Substance, and **b** Compound. *Light gray* structures clarify valence and connectivity. *Dashed lines* indicate aromatic bonds as perceived using the aromaticity model OEAroModelOpenEye in the OpenEye Scientific Software, Inc. OEChem C++ toolkit [57].

The total number of atom environments with radius *r* = 1 is 299,609 for Substance [100,411 (33.5%) singletons] and 109,306 for Compound [31,163 (28.5%) singletons], a 38× and 69×, respective, fold increase over *r* = 0. The distribution of fragment incidences is presented in Fig. 13. In both databases, respectively, only two atom environments with radius *r* = 1 occur in more than 50% of all structures. The top-10 most frequent atom environments with radius *r* = 1 in Substance and Compound are presented in Fig. 14. There is a clear indication that PubChem is dominated by structures containing aromatic fragments. While the atom environment consisting of three unbranched carbon atoms in a conjugated system is present in just above 50% of all structures (ranked second in both Substance and Compound), the corresponding branching atom environments are ranked first and third with examples shown in Fig. 14a (viii, ix) and b (viii, ix, x). In both Compound and Substance, the ten top-ranked atom environments consist of carbon, nitrogen and oxygen only.

**Fig. 15:**
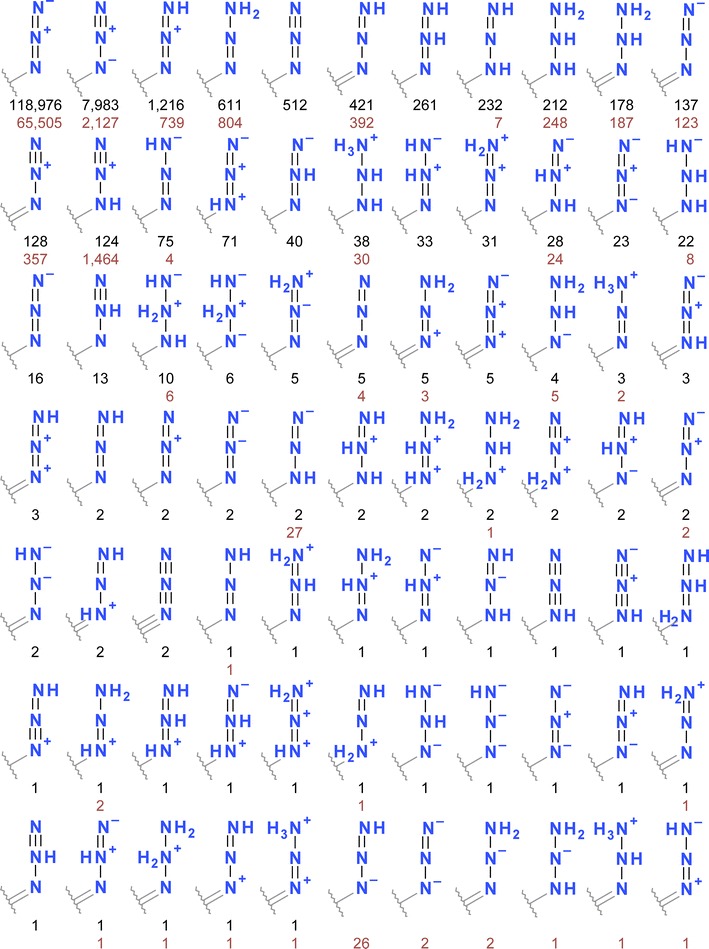
The 77 azide-like atom environments in PubChem. Fragment incidences are provided as absolute numbers. Configurations found in Substance are indicated by *black numbers* (71 variations), those present in Compound by *red numbers* (37 variations).

At least two functional groups are notorious for their variability and diverging preferred chemical structure representation: the azide and the nitro. To exemplify their heterogeneity, we performed a detailed analysis of the peculiarities of their basic element topologies present in PubChem. ‘Azide-like’ atom environments (‘*~N~N~N’; a di-coordinated nitrogen connected to a mono-coordinated and another di-coordinated nitrogen atom that is connected to some other atom) are found in Substance in 71 different variations of charge, hydrogen counts and connectivity as displayed in Fig. 15, of which 24 (34%) are singletons. The general definition of ‘azide-like’ groups retrieves numerous cases where it is unclear whether they were actually meant to represent an azide group. However, the two resonance structures of the azide functional group are the most frequent examples, occurring in 118,976 and 7,983 substances, respectively. The non-charge-separated representation is the fifth most frequent atom environment, occurring in 512 substances. In Compound, the number of atom environments matching the general pattern of an ‘azide-like’ group is reduced to 37 [11 (29.7%) singletons] with 6 new configurations that do not occur in Substance. Here, too, both resonance structures of the azide functional group exist, as normalization of azide functional group resonance structures was previously not handled by the PubChem structure standardization protocols. However, the non-charge-separated variant (fifth most frequent in Substance) is transformed to the most frequent atom environment of this type during PubChem standardization processing and therefore doesn’t exist in Compound.

**Fig. 16:**
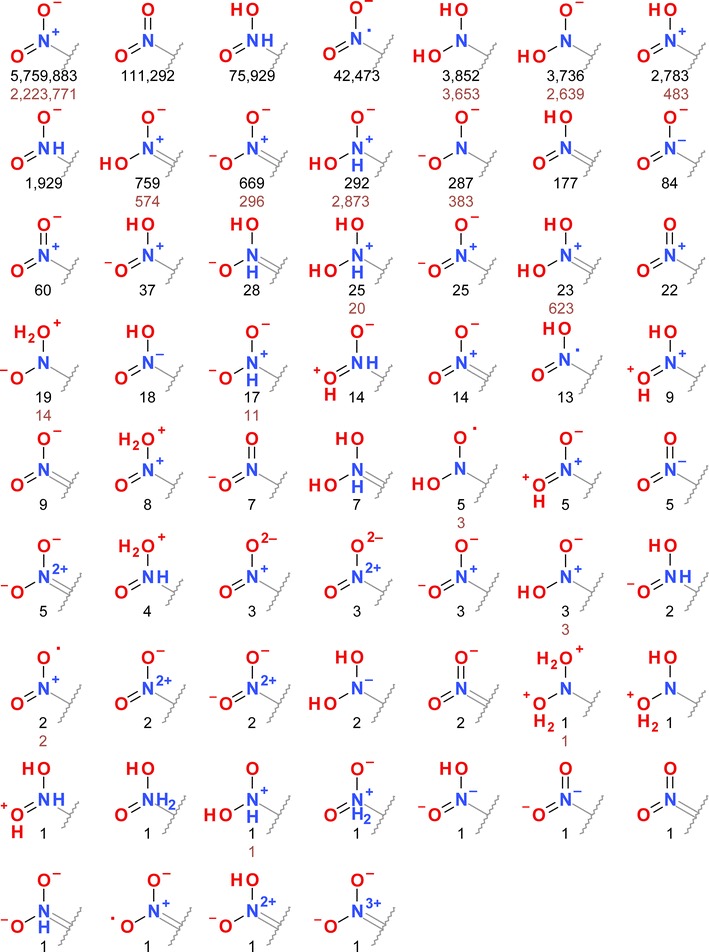
The 60 nitro-group-like fragments in PubChem. Fragments were obtained with the nitrogen atom as the central atom, ignoring the identity of the third adjacent atom. Fragment incidences are provided as absolute numbers. Configuration found in Substance are indicated by *black numbers*, those present in Compound by *red numbers*. Monoradicals indicated as *bullet*.

‘Nitro-group-like’ atom environments (‘*~N(~O)~O’; tri-coordinated nitrogen atom connected to two mono-coordinated oxygen atoms and some other atom) occur in 464 different variations of charge and connectivity, of which 148 (32%) are singletons. If the atom type of the fourth ‘other’ atom is ignored, the number of variations is reduced to 60 as displayed in Fig. 16, with 13 (22%) singletons. In Compound, this particular element topology occurs in 160 configurations [34 (21%) singletons], a number that is reduced to 17 variations (zero singletons) if the atom type of the fourth, not necessarily terminal, atom is ignored.

There are a number of reasons why, for a particular atom environment, the number of structures it is incident to varies between Substance and Compound. If Substance contains a number of duplicates of a structure, each duplicate contributes to the atom environment incidence; however, this redundancy is not present in Compound, as it contains only unique structures in terms of valence bond structure representations as normalized by PubChem. PubChem standardization can modify an atom environment, decreasing the number of times the pre-standardization environment is present in Compound, or even eliminating it in favor of an alternative, but equivalent, representation. If a Substance chemical structure contains invalid or erroneous atom environment configurations that cannot be salvaged by standardization, the corresponding substance structure is rejected and not present in Compound. Due to these effects, in general, the number of encountered atom environment variations and respective frequencies of occurrence will be reduced in Compound.

**Fig. 17:**
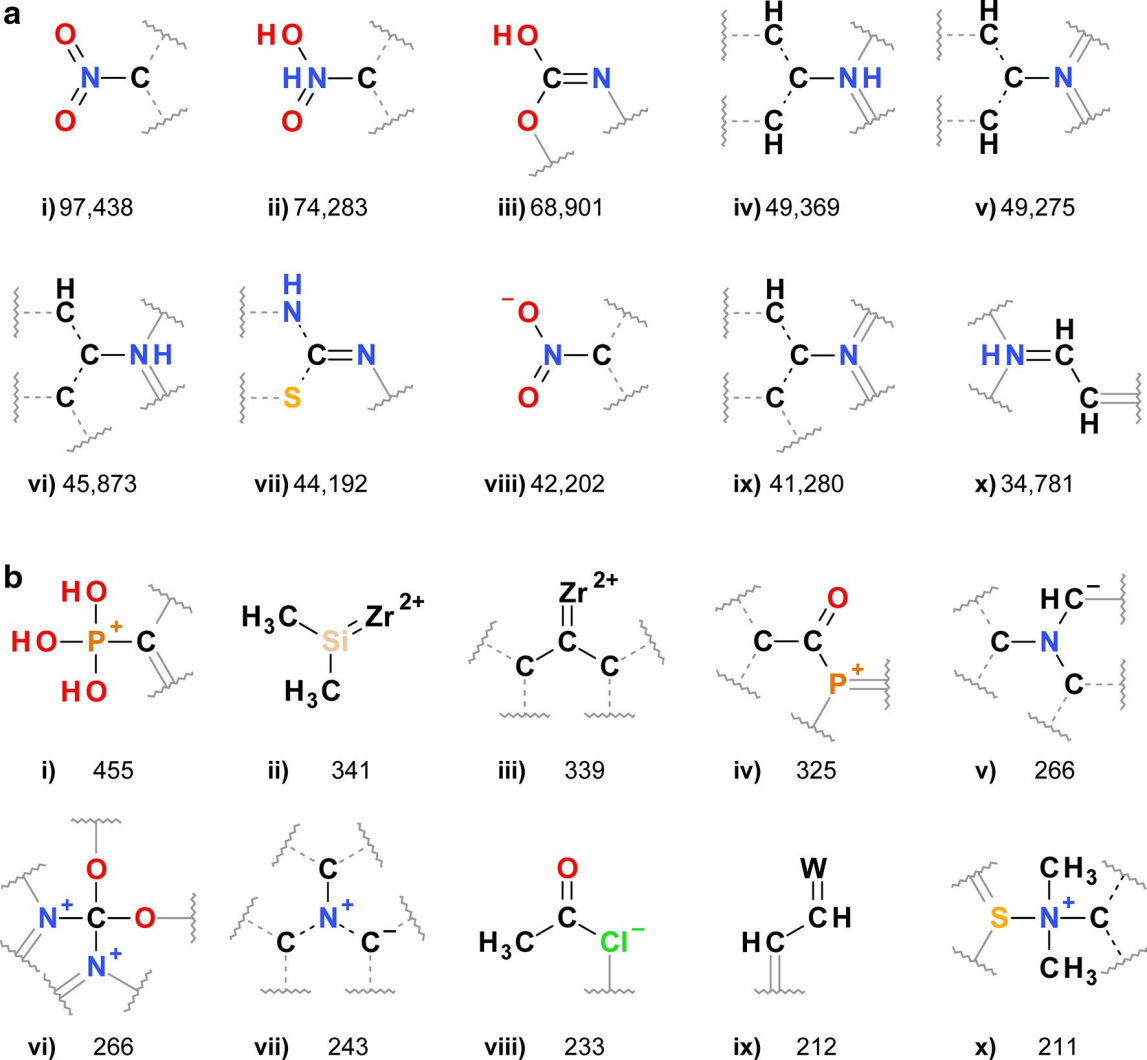
Top-10 atom environments with radius *r* = 1 unique to Substance and Compound, respectively. Atom environments are ranked by their incidence in the respective database: **a** Substance, and **b** Compound. Incidence provided in absolute numbers due to very low corresponding percentages. *Dashed lines* indicate aromatic bonds as perceived using the aromaticity model OEAroModelOpenEye in the OpenEye Scientific Software, Inc. OEChem C++ toolkit [57].

In total, 203,232 atom environments with radius *r* = 1 occur in Substance but not in Compound, of which 81,702 (40.2 %) are singletons. The top-10 most frequent occurring of these atom environments are shown in Fig. 17a. The Substance unique atom environments of rank one, two and eight are instances of the generalized ‘nitro-group-like’ environments that are modified to the preferred representation of this functional group. The third and seventh most frequent (*r* = 1) atom environments unique to Substance are examples of modifications occurring during structure standardization that are due to tautomeric preference: the “[OH]C=N” pattern is modified to “O=C[NH]”, and the “[NH]–:C=N” pattern to “N=:C–[NH]” (where ‘–:’ indicates an aromatic single bond and ‘=:’ indicates an aromatic double bond). In the case of the atom environments ranked fourth, sixth, and tenth, depending on whether all indicated bonds actually represent covalent bonds, either the implicit hydrogen atom on the nitrogen atom is replaced by a positive charge, or it is removed when the further outgoing single bond is replaced by a non-standard bond type. Environments of ranks five and nine are related to the top ranked representation of the nitro group in diverging molecular contexts and reflect modifications to the respective nitrogen atom after standardization.

There are 12,929 atom environments with radius *r* = 1 in Compound that do not occur in Substance, of which 6,134 (46.6%) are singletons. Consequently, all of them are a result of the PubChem structure standardization protocols. The top-10 most frequent examples are presented in Fig. 17b. In most cases, they are due to simple atom type modifications. In the most frequent and fourth most frequent case, tetra-valent phosphorous that was deposited uncharged gets assigned a positive charge. The charges on zirconium in the second and third most frequent cases are the result of attempts to find an adequate representation of complex bonds the respective atoms are involved. In a similar fashion, this is how the negative charges on carbon atoms in the fifth and seventh example occur. A positive charge is placed on tetra-valent nitrogen that was deposited uncharged in the cases ranked sixth, seventh and tenth. The di-valent chlorine atom showcased in the eighth most frequent environment exclusively found in Compound was deposited without charge and is modified to erroneously standardize to this atom type. In the substances that correspond to the ninth most frequent example, the tungsten atom is part of a complex with covalent single bonds that were removed during standardization by converting them to a PubChem complex bond, yielding a new atom type and consequently a new atom environment.

The full list of atom environments with radius *r* = 1 with their respective incidence is provided for Substance and Compound as supporting information in Additional file 4.

### Atom environments radius r = 2

**Fig. 18:**
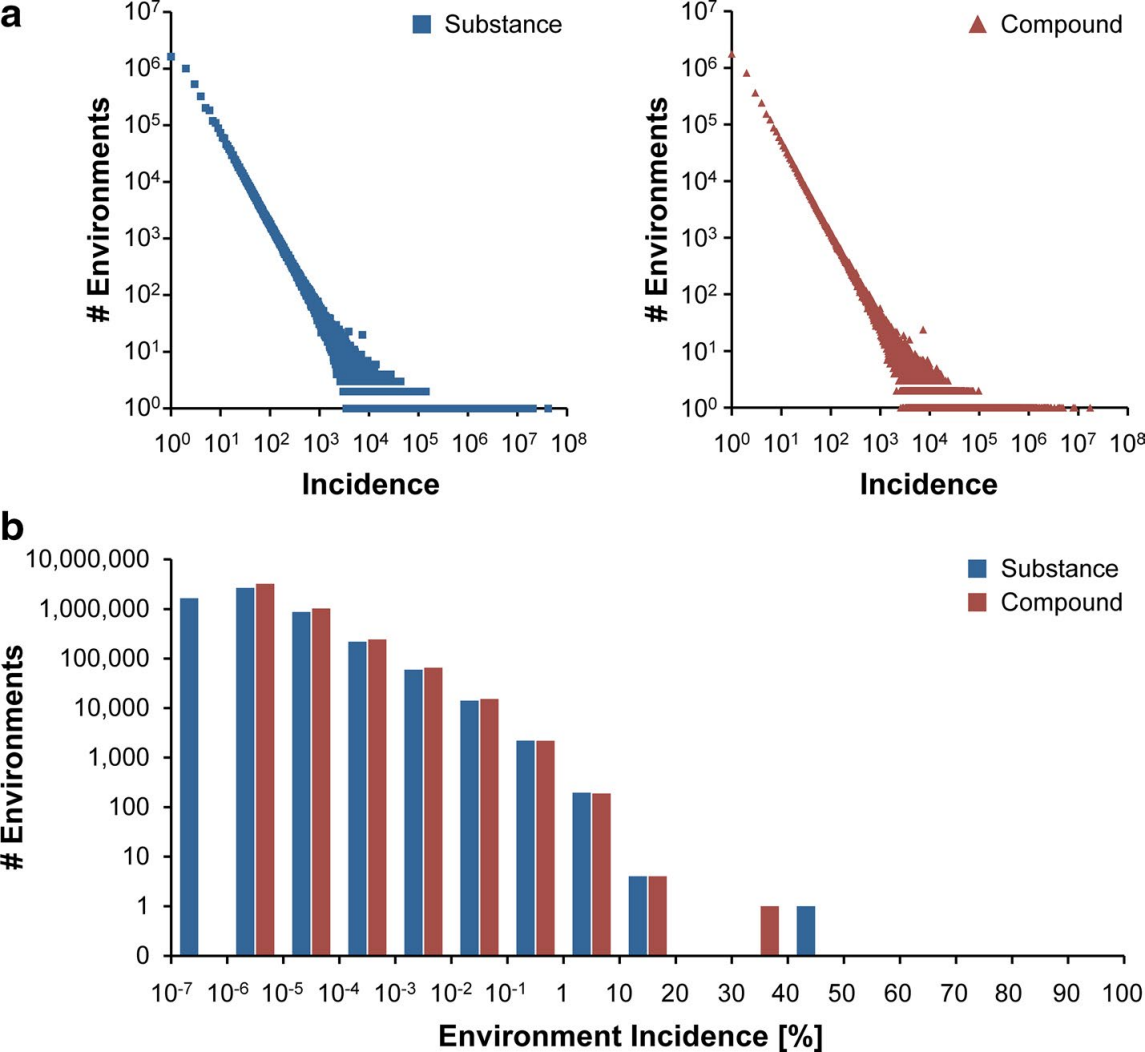
Atom environment statistics radius *r* = 2. **a** Distribution of atom environment incidences (log–log scale plots); **b** Histogram of incidence percentages; minima inclusive, maxima exclusive; range from 10^−7^ to 10% logarithmic and linear 10–100% for clarity.

**Fig. 19:**
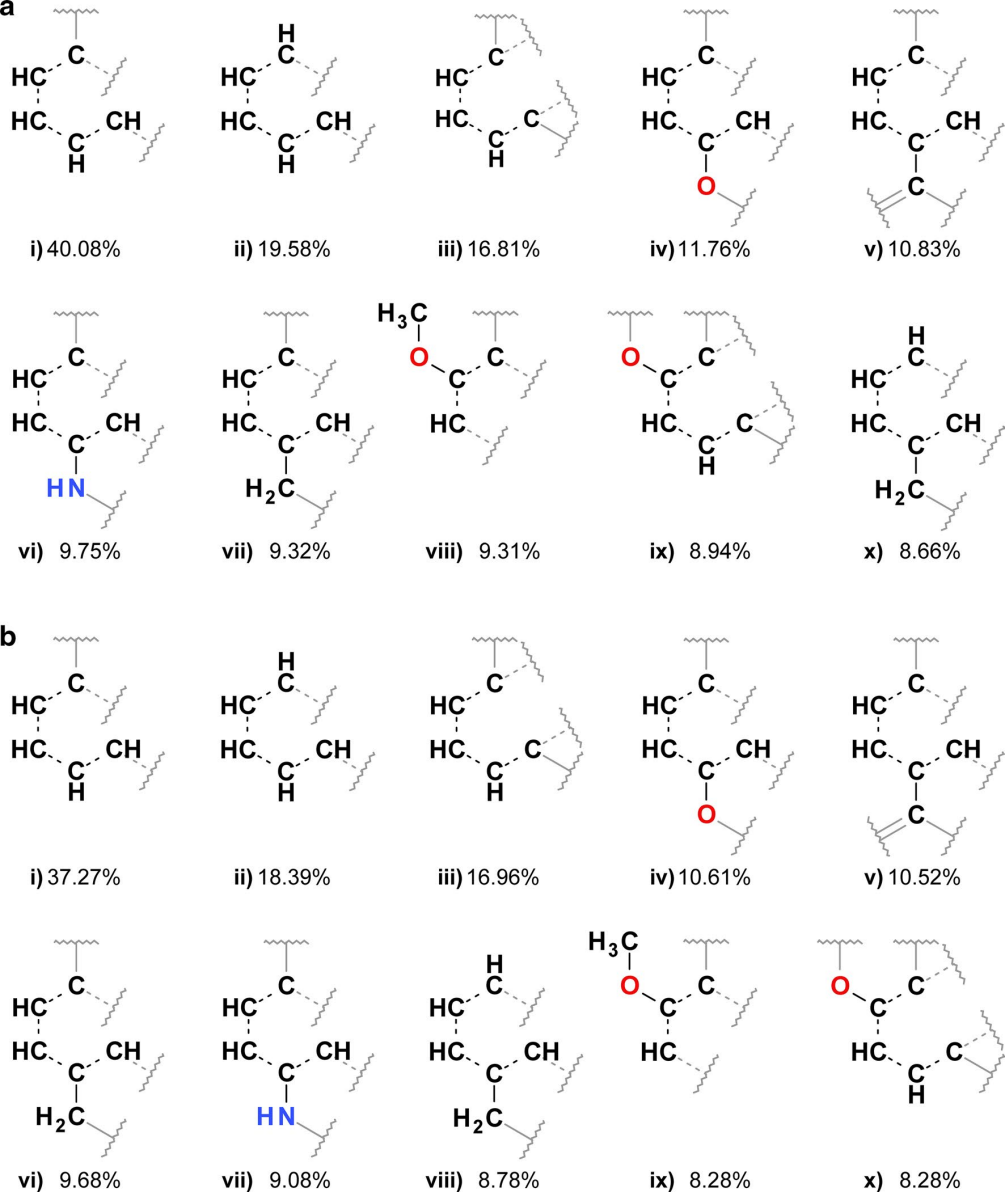
Top-10 most frequent atom environments with radius *r* = 2 in PubChem. Percentages indicate incidence of the atom environment in the respective database: **a** Substance; and **b** Compound. *Light gray structures* clarify valence and connectivity. *Dashed lines* indicate aromatic bonds as perceived using the aromaticity model OEAroModelOpenEye in the OpenEye Scientific Software, Inc. OEChem C++ toolkit [57].

The number of unique atom environments with radius *r* = 2 is 5,453,889 in Substance (1,637,544 [30%] singletons) and 4,559,587 in Compound (1,782,077 [39.1%] singletons), an 18× and 42×, respective, fold increase over *r* = 1. The distribution of fragment incidences is presented in Fig. 18. Unlike with *r* = 1, and emphasizing the diversity of structures when more atoms are considered, no fragment occurs in more than 50% of the structures in either Substance or Compound. The respective top-10 most frequent atom environments, presented in Fig. 19, are identical between Substance and Compound; however, the order is varied. In Substance, the most frequent atom environment occurs in 40.1% of all substances. The same fragment occurs in 37.3% of all compound records. In general, the top-ranked atom environments at the *r* = 2 level are the logical extensions to the most frequent atom environments at the *r* = 1 level, and, therefore, have a substructure-superstructure relationship to those shown in Fig. 13. For example the atom environments in Fig. 19b (i–iii) (*r* = 2, PubChem Compound) are combinations of those generated with a smaller radius, shown in Fig. 13b (i) and (ii) (*r* = 1, PubChem Compound). Superstructures to the non-aromatic environment with radius *r* = 1 are not in the top-10 most frequent environments with radius *r* = 2. Evidently, the frequency of aromatic fragments is sufficient such that variations including the next sphere of atoms can be found in more structures than the most frequent non-aromatic atom environments. This can be observed when comparing the incidences of the most frequent environments with radius *r* = 1 and *r* = 2.

**Fig. 20:**
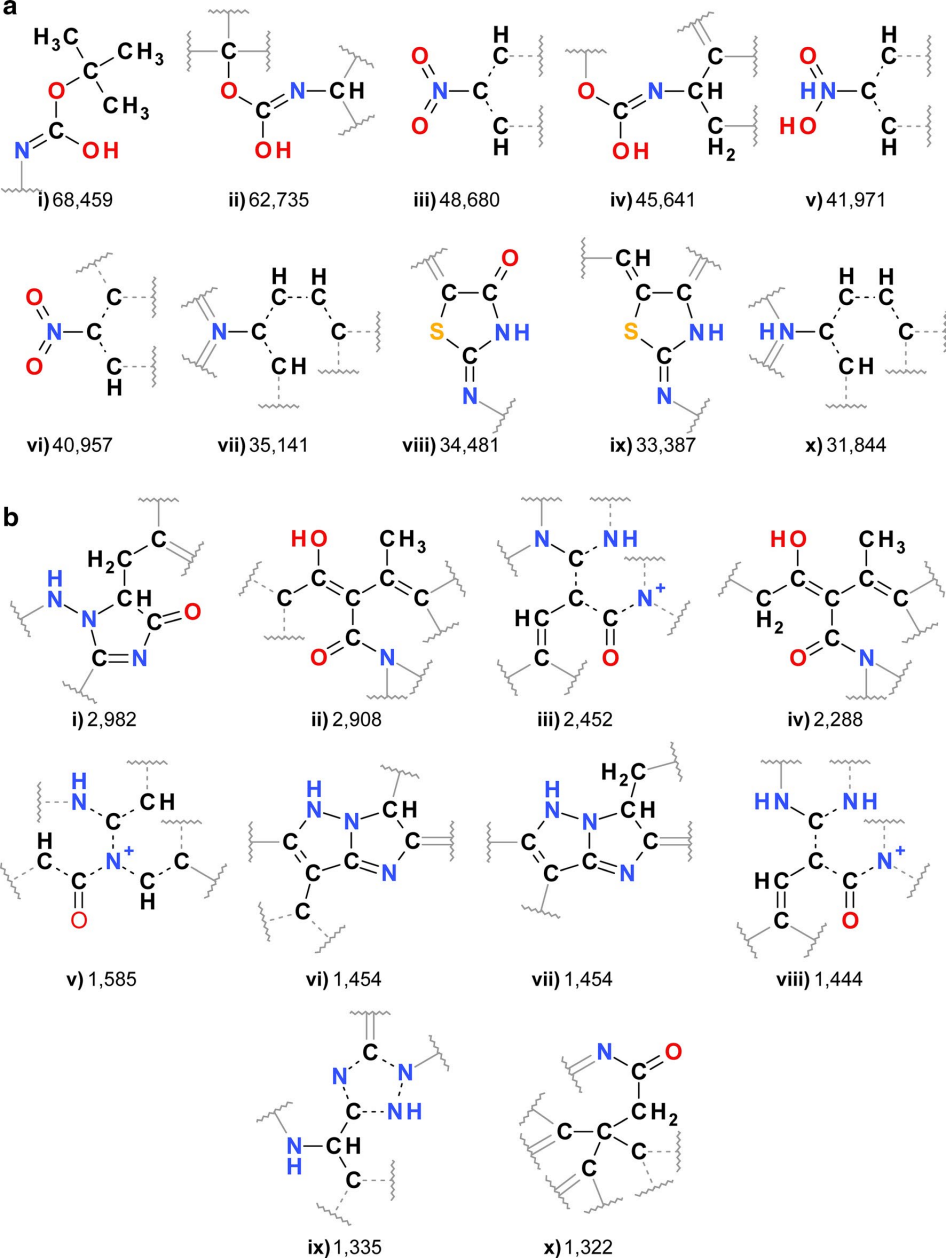
Top-10 most frequent atom environments with radius *r* = 2 unique to Substance and Compound, respectively. Atom environments are ranked by their incidence in the respective database: **a** Substance; and **b** Compound. Incidence provided in absolute numbers due to very low corresponding percentages. *Dashed lines* indicate aromatic bonds as perceived using the aromaticity model OEAroModelOpenEye in the OpenEye Scientific Software, Inc. OEChem C++ toolkit [57].

The top-ranked environments present in Substance but not in Compound (Fig. 20a) can be explained by two effects. One is the standardization of the nitro group, responsible for unitizing the representations indicated in cases (iii), (v), (vi), (vii) and (x). The second one is a structural modification due to tautomer standardization procedures. In the cases shown in Fig. 20a (i) and (ii), which are closely related and actually possible extensions of each other, originating from diverging central atoms, the “N=C–OH” fragment gets standardized to the keto-equivalent “HN–C=O”. The same applies to rank (iv). For ranks (viii) and (ix), the “N=C–NH” pattern is modified to “HN–C=N” such that the double bond is in a ring. For the top-ranked atom environment found in Compound but not in Substance is provided in Fig. 20b (i), the modification occurring during standardization actually takes place partly outside of the atom environment, but can be seen in its entirety in the cases shown in Fig. 20b (vi) and (vii), where the complete tautomeric system is found in the atom environment. It depicts a 1,5-shift of a proton between ring nitrogen atoms. In the case of the atom environment in Fig. 20b (ii), the hydrogen atom was originally located on the other oxygen atom (another proton 1,5-shift). Atom environments shown in Fig. 20b (iii, v) and b (viii) are examples of simpler modifications resulting from the application of standardization protocols. For example, the positive charge on the nitrogen atom resulted from fixing a penta-valent nitrogen atom. The atom environment shown in Fig. 20b (iv) is analogous to Fig. 20b (ii). In the case of atom environment Fig. 20b (ix), a proton on a ring nitrogen atom is transferred in a 1,3-shift to another ring nitrogen atom. In the case of atom environment Fig. 20b (x), the nitrogen atom type is changed during standardization from tri-valent, tri-coordinated with one implicit hydrogen atom to tri-valent, di-coordinated with no implicit hydrogen atom due the proton being shifted to an atom outside of the environment.

The full list of atom environments with radius *r* = 2 with their respective incidence is provided for Substance and Compound as supporting information in Additional file 4.

### Atom environments radius r = 3

**Fig. 21:**
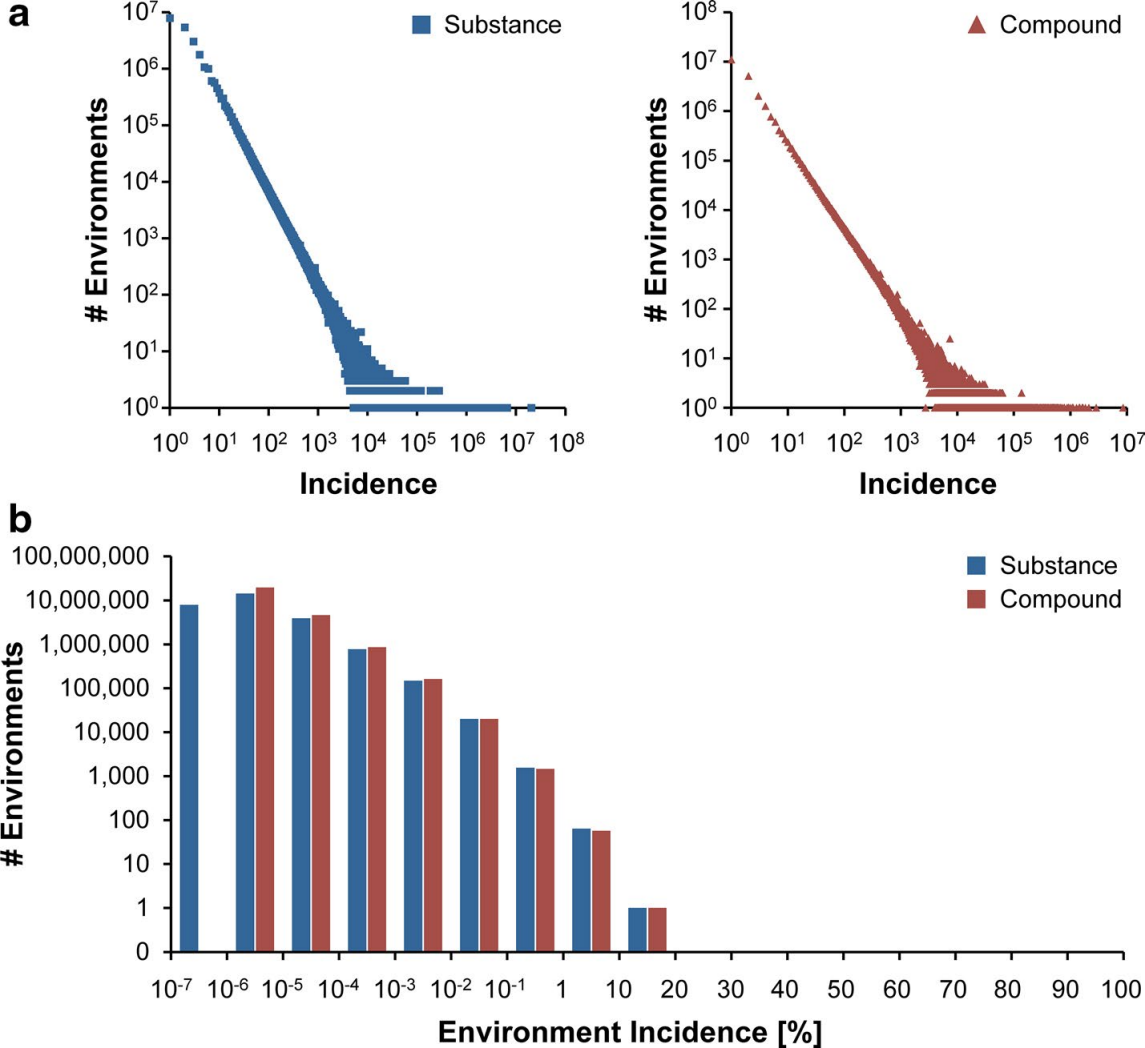
Atom environment statistics radius *r* = 3. **a** Distribution of atom environment incidences (log–log scale plots);** b** Histogram of incidence percentages; minima inclusive, maxima exclusive; range from 10^−7^ to 10% logarithmic and linear 10–100% for clarity.

**Fig. 22:**
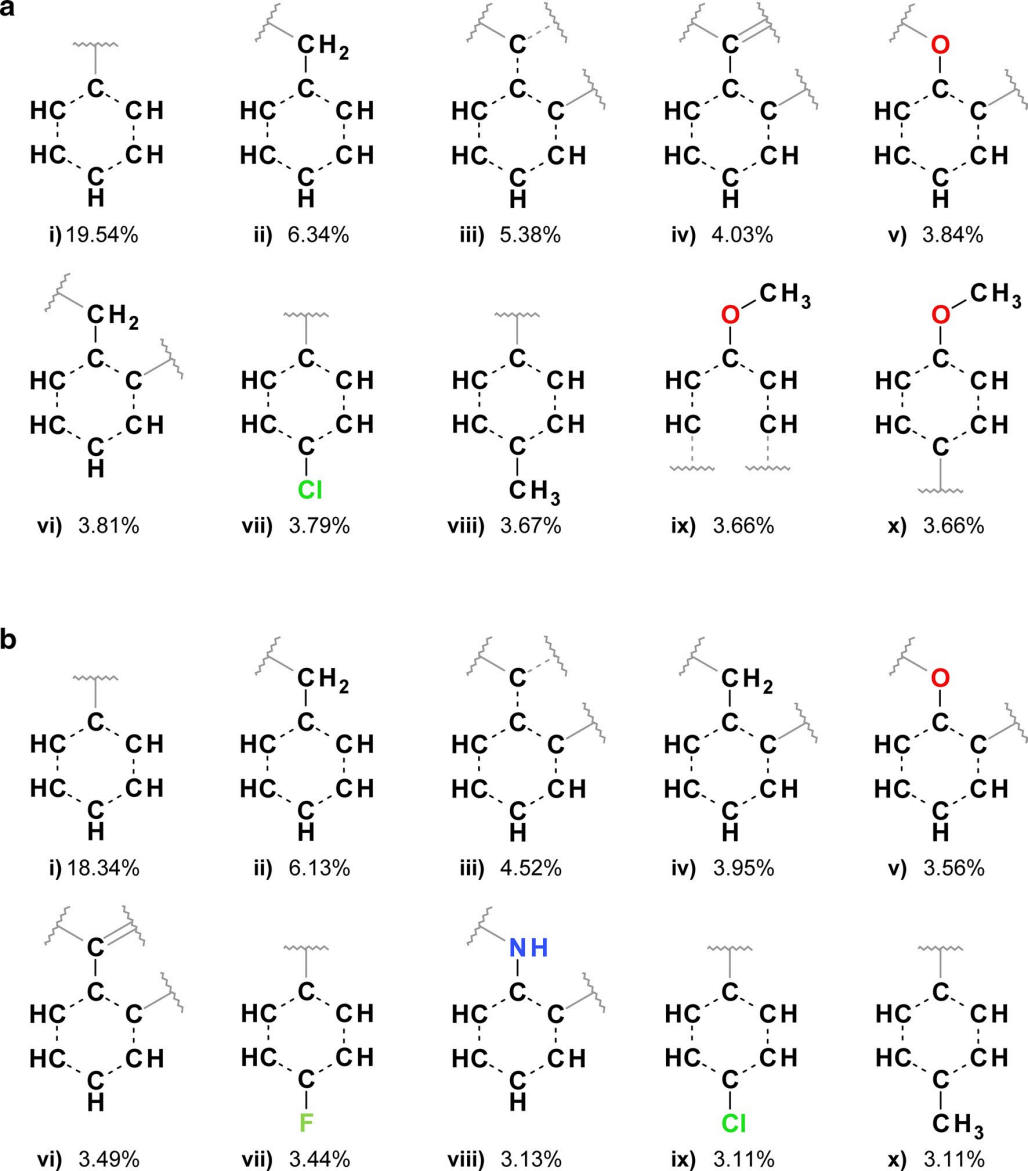
Top-10 most frequent atom environments with radius *r* = 3 in PubChem. Percentages indicate incidence of the atom environment in the respective database: **a** Substance; and **b** Compound. *Light gray* structures clarify valence and connectivity. *Dashed lines* indicate aromatic bonds as perceived using the aromaticity model OEAroModelOpenEye in the OpenEye Scientific Software, Inc. OEChem C++ toolkit [57].

The number of unique atom environments with radius *r* = 3 is 26,988,962 in Substance (7,879,785 singletons, 29.2%) and 25,115,177 environments in Compound (11,074,304 singletons, 44.1%), a 4.9× and 5.5×, respective, fold increase over *r* = 2. The distribution of fragment incidences is presented in Fig. 21. The top-10 most frequent atom environments are presented in Fig. 22. The most frequent environment occurs in 19.5% of all substances and 18.3% of all compounds, respectively. In Substance, the most frequent atom environment with radius *r* = 3 is a benzene-ring with only one substituent/attachment point (19.5% incidence). In the second most frequent Substance atom environment (*r* = 3), this attachment is specified as a tetra-valent tetra-coordinated carbon atom with two implicit hydrogen atoms that is uncharged and not part of an aromatic moiety (6.3% incidence). It is a superstructure of the first ranked environment, the two differ by the center atom. Substance atom environments (*r* = 3) ranked third, fourth, fifth and sixth show examples with two substituents in the ortho position, where only in the case of the third ranked environment a connection to another aromatic system is indicated. In respective order of their ranks, these substituents are uncharged, aromatic, tri-coordinated and tetra-valent carbon with no implicit hydrogen atoms (5.4% incidence), uncharged, tri-coordinated and tetra-valent carbon with no implicit hydrogen atoms (4% incidence), uncharged, di-coordinated and di-valent oxygen with no implicit hydrogen atoms (3.8% incidence), and uncharged, di-coordinated and tetra-valent carbon with two implicit hydrogen atoms (3.8% incidence). For these cases, the second substituent is not further specified, due to the location of the center atom of the environment; naturally, these generalized cases are more frequent than the more discriminative fragments specifying this part as well. The environments ranked seventh and eighth show examples for para substituted benzene rings, with one substituent being specified with only a single terminal chlorine atom (ranked seventh, 3.8% incidence) or a methyl group (ranked eighth, 3.7% incidence). The ninth- (3.7% incidence) and tenth-ranked (3.7% incidence) environments are closely related to the one with radius *r* = 2 shown in Fig. 19a (iv), further specifying the substituent, and, in the case of the tenth-ranked, the sixth carbon atom in the phenyl ring.

The top-10 most frequent environments (*r* = 3) in Compound are almost identical to those in Substance. The three top-ranked fragments are the same. Between Substance and Compound, the fourth and sixth ranked environment switched ranks. Instead of a para substituted benzene ring with a terminal chlorine atom as one of the substituents, the fluorine case is more frequent in Compound and consequently ranked lower with 3.4% incidence (fluorine, ranked seventh) compared to 3.1% incidence (chlorine, ranked ninth). The eighth ranked atom environment (*r* = 3) in Compound is not among the top-10 in Substance, with a tri-valent, tri-coordinated and uncharged nitrogen atom with one implicit hydrogen atom in ortho position to a second substituent in the benzene ring (3.1% incidence). Lastly, the fragment with a methyl group in para position to a second unspecified substituent (3.1% incidence) is ranked tenth in Compound compared to eighth in Substance.

**Fig. 23:**
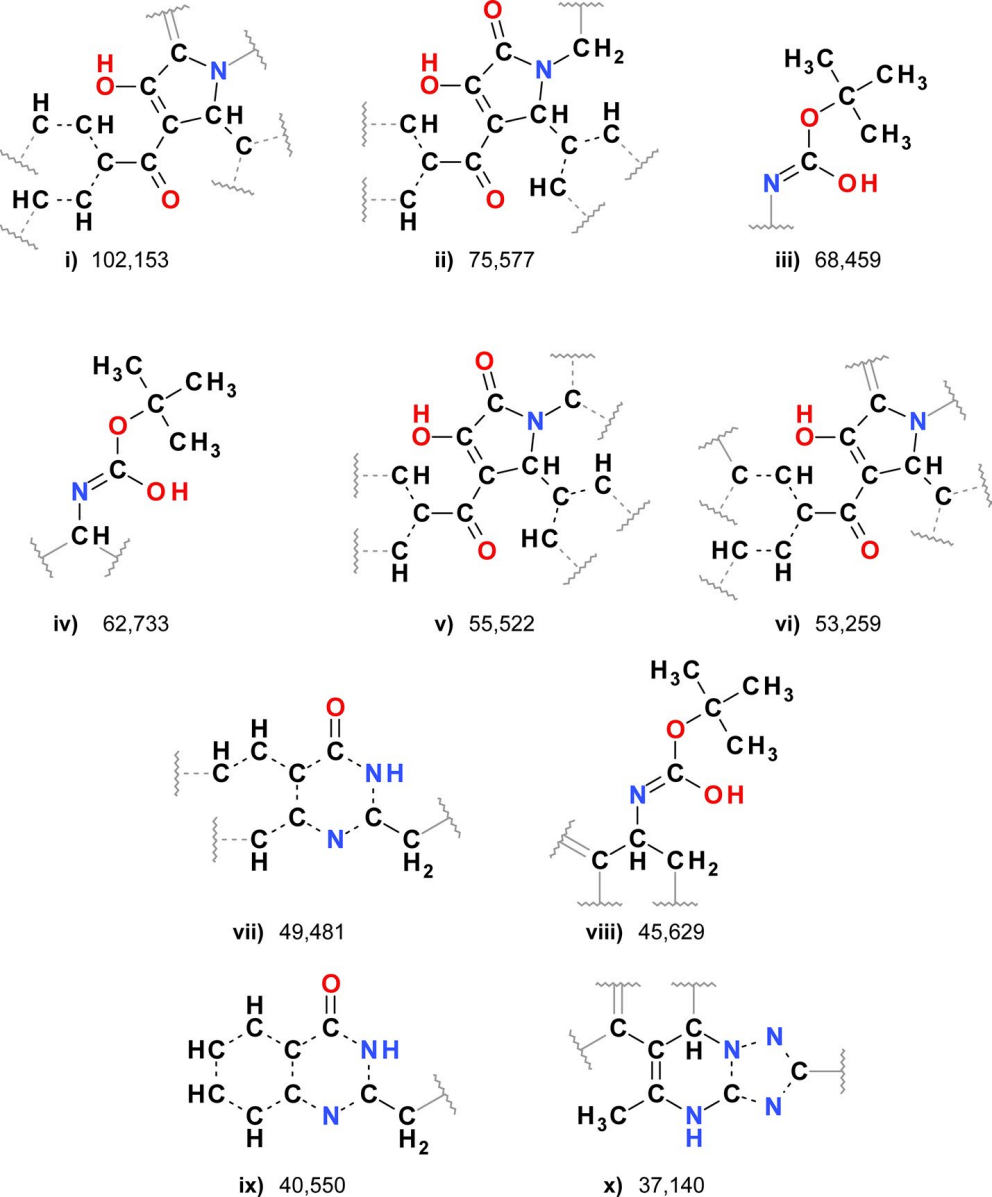
Top-10 most frequent atom environments with radius *r* = 3 unique to Substance. Atom environments ranked by incidence. Incidence provided in absolute numbers due to very low corresponding percentages. *Dashed lines* indicate aromatic bonds as perceived using the aromaticity model OEAroModelOpenEye in the OpenEye Scientific Software, Inc. OEChem C++ toolkit [57].

The first and second most frequent atom environments (*r* = 3) that are found in Substance but not in Compound (Fig. 23) are closely related and show almost identical substructures originating from different central atoms. In both cases, during standardization, the proton from the exocyclic hydroxyl group is transferred in a 1,3-shift to the corresponding carbon atom, in the same step moving the double bond out of the five-membered ring. It is noteworthy that without the inclusion of all connecting bonds in the environment in the fragmentation model, the top-ranked environment is an example where the five-membered ring would not be closed. The next two cases, three and four, are similar as well, converting the “HO–C=N” fragment to “O=C–NH”. Cases five and six are again variations of the first two examples, one time with a different center atom than the environment ranked first, and the other time with a different aromatic carbon atom type. Fragments ranked seventh and ninth have different central atoms but exhibit the same 1,3-shift of a proton between nitrogen. The environment ranked eighth follows the same pattern as ranks three and four. In the last example listed in Fig. 23, during standardization the proton on the nitrogen atom is transferred in a 1,5-shift to its counterpart depicted at the top of the five-membered ring.

**Fig. 24:**
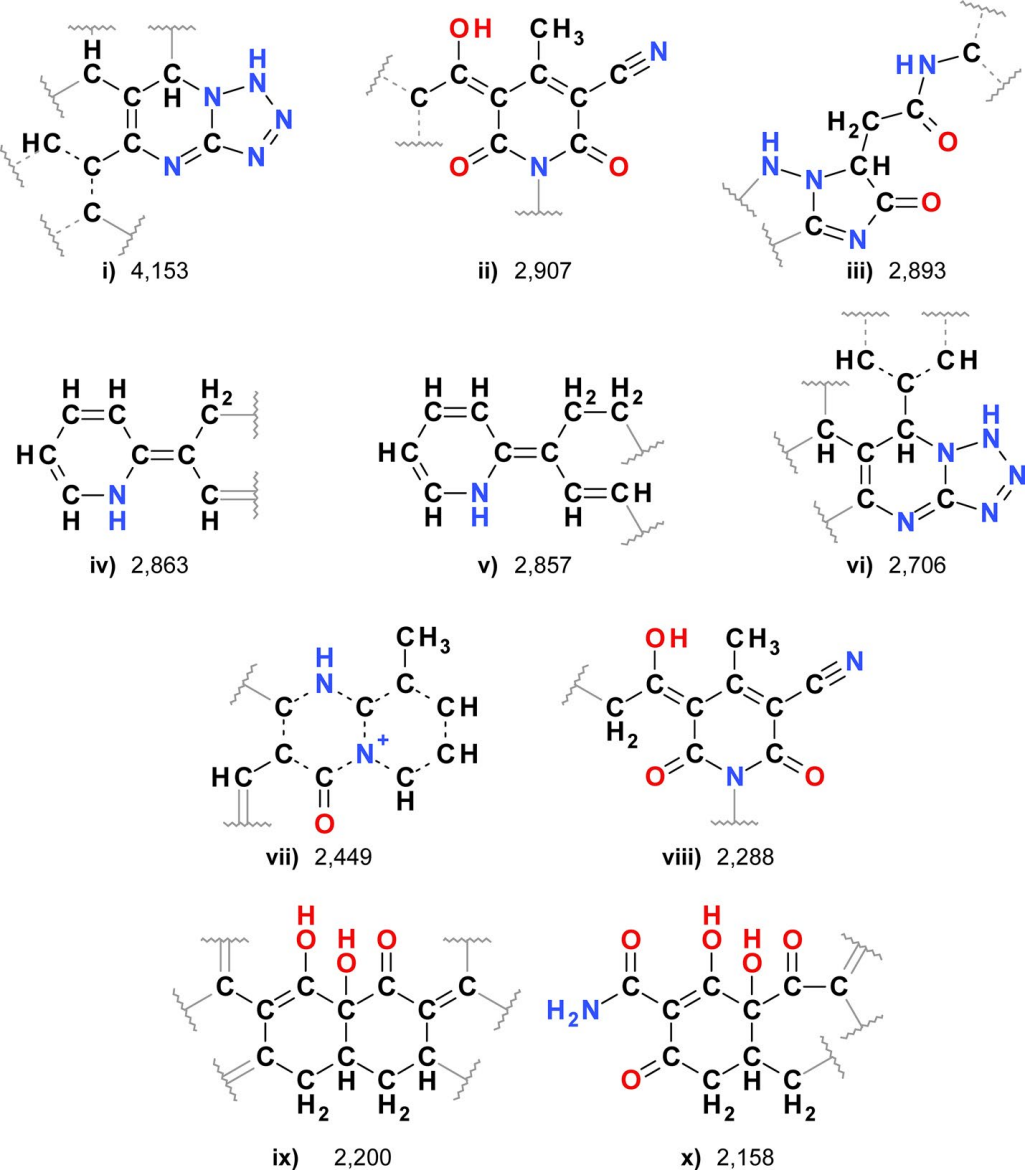
Atom environments with radius *r* = 3 unique to Compound. Atom environments ranked by incidence. Incidence provided in absolute numbers due to very low corresponding percentages. *Dashed lines* indicate aromatic bonds as perceived using the aromaticity model OEAroModelOpenEye in the OpenEye Scientific Software, Inc. OEChem C++ toolkit [57].

The atom environments (*r* = 3) identified to be present in Compound but not Substance (Fig. 24) are results of the PubChem standardization protocols. For the fragments ranked first and sixth, the deposited structure had the proton located on the nitrogen atom in the six-membered ring rather than the five-membered ring. The environments ranked second and eight similarly handle a case whereby the double bonded oxygen closest to the hydroxyl group originally was specified as hydroxyl group and the proton was moved during standardization (double bond changes implied). In the third ranked environment, analogous to the case shown in Fig. 20b (i) with *r* = 2, a proton shift occurs that involves atoms in the original structure that are not part of this particular atom environment. Originally, the proton was located on the nitrogen atom that ends up being double bonded in the five-membered ring. Cases four and five involve an exocyclic double bond where the double bond was originally in the six-membered ring, showing a PubChem preference to put the tautomeric mobile proton on the nitrogen atom. The seventh-ranked environment is an example of a simple atom type modification, where the positively charged nitrogen atom was originally deposited uncharged with an additional (implicit) hydrogen atom attached. The last two (related) cases exhibit one completely observable modification and one that involves structural elements outside of the depicted fragment. The exocylic double bonded oxygen atom in the last case was deposited as hydroxyl group, but its proton got moved to the oxygen atom resulting as the hydroxyl group attached to the carbon atom that is part of the six-membered ring (double bond changes implied).

The full list of atom environments with radius *r* = 3 with their respective incidence is provided for Substance and Compound as supporting information in Additional file 4.

### Atom environment set overlap

**Fig. 25:**
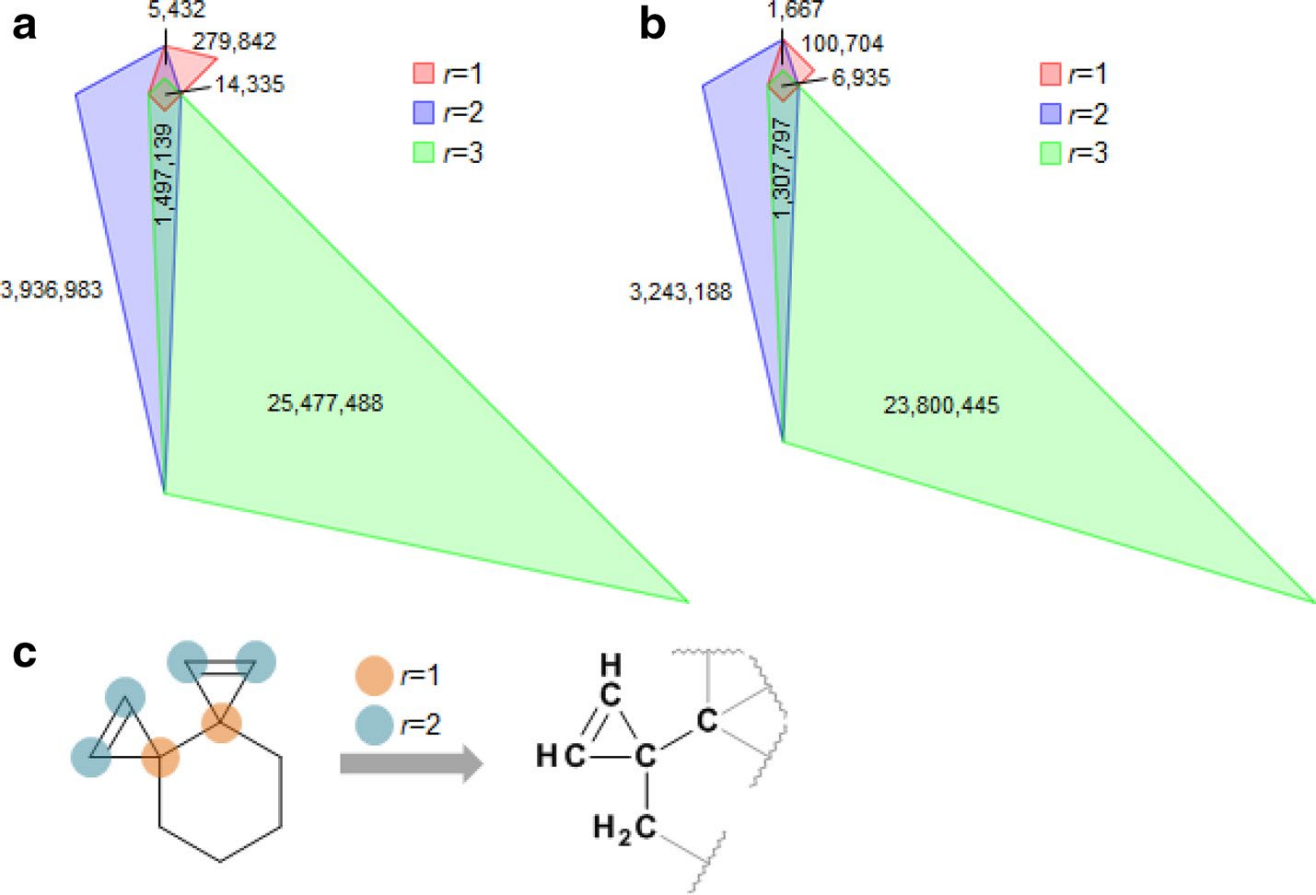
Atom environment set overlap. The area-proportional Venn diagrams illustrate how the sets of atom environments obtained with radii *r* = 1,2,3 overlap for the respective database. **a** Substance, **b** Compound, **c** Example from dispiro(2.0.2.4)deca-1,5-diene (CID 143166) illustrating how environments obtained with radius *r* = 1 (center atom indicated in *orange*) and radius *r* = 2 (center atom indicated in *teal*) can be identical.

The sets of atom environments with radii *r* = 1, *r* = 2 and *r* = 3 partly overlap, as illustrated in the Venn diagrams shown in Fig. 25. This overlap occurs for two reasons. First, depending on the central atom and the molecular graph, starting from different atoms can result in the same atom environment with different radii. This is illustrated in Fig. 25c, using the example of dispiro(2.0.2.4)deca-1,5-diene (CID 143166). Second, atom environments can cover entire structures and consequently cannot grow any further with higher order radii. The second case is more prominent than the first one, with 13,918/43,575/916,758 environments with radius *r* = 1/*r* = 2/*r* = 3 describing complete structures in Substance, respectively. The corresponding numbers for Compound are 6,589 (*r* = 1), 31,804 (*r* = 2) and 838,487 (*r* = 3). Naturally, there is no overlap between *r* = 1 and *r* = 3 environments exclusively. Such fragments would have to cover the entire chemical structure at *r* = 1 level and therefore also be part of the set of environments generated for *r* = 2. The top-10 most frequent cases for every radius in Substance and Compound are supplied as supporting information in Additional file 1: Figures S2 and S3.

### Atom environment rate of growth

This atom environment survey of PubChem content is revealing for multiple reasons. One potential surprise is the high rate of singletons in Substance and Compound as a function of atom environment radius. The percentage of atom environment singletons for Substance is nearly constant, being 32.5, 33.5, 30, and 29.2% for radius *r* = 0, 1, 2, and 3, respectively. For Compound, however, the percentage of atom environment singletons is steadily increasing, being 10.6, 28.5, 39.1, and 44.1% for radius *r* = 0, 1, 2, and 3, respectively. Furthermore, the rate of growth of new atom environments in both Substance and Compound slows dramatically as a function of increasing radius, with an increase of 69, 42, and 6 times for Compound and 38, 18, and 5 times for Substance when considering the ratio of atom environment radius 0–>1, 1–>2, and 2–>3, respectively. The increasing quantity of singletons and the decreasing rate of growth of atom environments as a function of atom environment radius implies that there is still an enormous chemical space to explore even at the chemical fragment level, with nearly half of all *r* = 3 molecular fragments in Compound appearing in only a single structure. It may also be surprising or revealing (to some) that the count of unique atom environments in Compound is so relatively few, being 1,583 (*r* = 0), 109,306 (*r* = 1), 4,559,587 (*r* = 2), and 25,115,177 (*r* = 3). On the other hand, the drop in increase of number of fragments from *r* = 1 to *r* = 2 compared to r = 2 to *r* = 3 could also indicate that there are constraints limiting a full combinatorial exploration of the space defined by smaller fragments (e.g., steric effects). Furthermore, an increasing number of functional groups results in more reactive structures that would be increasingly harder to synthesize.

The high rate of singletons suggests that sampling a database for atom environments, such as in the earlier 1971 study, will miss the vast majority of atom environments present; however, sampling should be sufficient to locate common fragments. Therefore, the rate of growth of ‘augmented atoms’ determined in this study of 7.9 from 1971 CASRS to present day PubChem should be considered an upper bound due to the use of structure sampling in the 1971 CASRS study. Without the full CASRS database for comparison, a more accurate determination of rate of growth cannot be determined.

The concept of circular atom environments is the basis for so-called radial/circular [32, 33], Morgan [56], or extended connectivity fingerprints (ECFP) [34]. As feature ensemble fingerprints, they are not based on a predefined dictionary of structural features. However, we identified in total 28,460,736 unique atom environments of radii *r* = 1, *r* = 2 and *r* = 3 (of which 12,470,387 are singletons, 43.8%) in addition to 1,583 atom types (*r* = 0, 167 singletons, 10.6%) in PubChem Compound, in total close to 2^25^ fragments. For the particular example of ECFP, where a hash function is used to generate 32-bit integers as identifiers for circular fragments [34], more than 99% of the available 2^32^ (4,294,967,296) bits in a fingerprint may remain unused. In Compound, the number of observed singletons increases with increasing radius, from 11% for atom types (*r* = 0), 29 % at *r* = 1, 39 % at *r* = 2, to 44 % at r = 3. It is unclear how well these singular differences between structures are captured in fixed size structural keys (such as those used by PubChem [72]). Conversely, as dynamically generated fingerprints like ECFP quantify structural differences including those previously unknown, this might be a possible explanation for their general advantage over other fingerprint approaches. Further analysis of molecular fragments, such as those provided in this study, may prove useful to design a better a set of discriminating (i.e., not co-linear) structural patterns useful for cheminformatic purposes such as structure searching (e.g., identity, substructure, similarity, etc.), virtual screening, clustering, clique analysis, and other conceivable applications.

### Atom environment errors

**Fig. 26:**
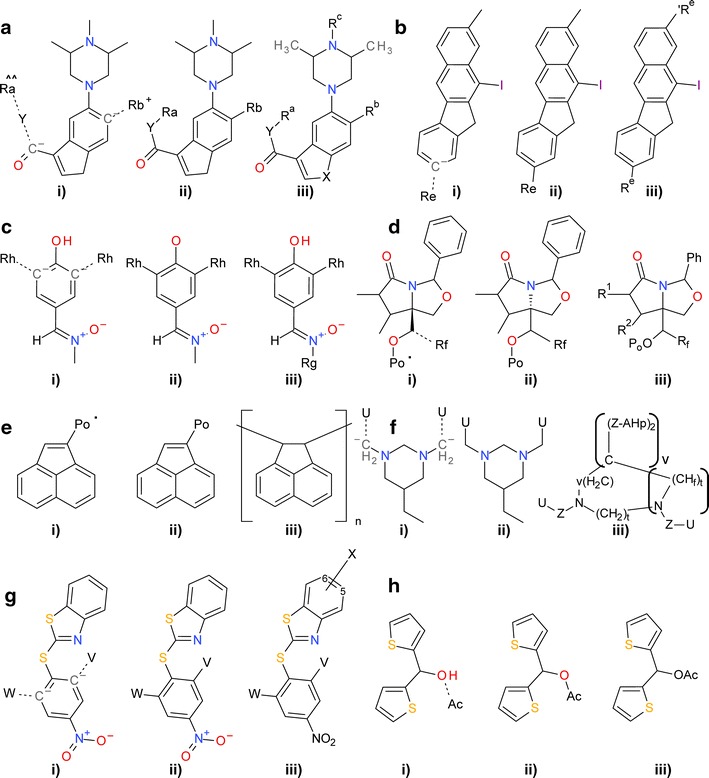
Sources of some erroneous atom environments in PubChem. **a** Radium (Ra), rubidium (Rb) and yttrium (Y), **b** Rhenium (Re), **c** Rhodium (Rh), **d** Rutherfordium (Rf) and polonium (Po), **e** Polonium (Po), **f** Uranium (U), **g** Vanadium (V) and tungsten (W), **h** Actinium (Ac). In all cases: *i* as present in Compound, *ii* as deposited in Substance, *iii* as in the original context, except for (E), where it describes a correct way to annotate the ‘polymer’ aspect of the intended structure ‘polyacenapththylene’. Charges and radical annotation (• doublet monoradical, ^^ triplet diradical) in *i* are a result of the PubChem standardization protocols. *Dashed bonds* indicate PubChem non-standard bonds.

This survey describes the content of PubChem and, as a consequence, has to deal with and is affected by its idiosyncrasies that can lead to an overestimation of atom types and, therefore, atom environments as well. Beyond automated checking by algorithms, there is no manual curation of data in PubChem, as it is not practical to curate tens of millions of chemical records; however, PubChem contains many manually curated chemical collections. Information is deposited into Substance and subjected to automated cleanup and standardization protocols, and the resulting standardized structures are the content of Compound. This only means that they are considered to be ‘valid’ chemistry, but not necessarily ‘real’ or ‘correct’ in terms of the intended structure. One can find cases where a ‘valid’ structure is in fact incorrect. For example, this can occur whenever something is mistaken for chemical information that has a different meaning in the original context. As shown in Fig. 26a, CID 60023123 contains radium (Ra), rubidium (Rb) and yttrium (Y) atoms. The structure, deposited as SID 142797524, originated from the USPTO patent application US2005165025. The Ra, Rb, and Y ‘atoms’ are actually placeholders to define larger moieties and are not really elements. From the same patent structure, the literals ‘X’ and ‘Rc’, neither found in the periodic table, are not provided in this substance—instead they are provided to PubChem as carbon atoms. The same issue can be found with other literals used as placeholders in patents, but that also exist in the periodic table. In Fig. 26b, the case of rhenium (Re) is illustrated with CID 60092055, found in SID 143170472 originating from USPTO patent US7335659. Rhodium (Rh) atoms can be the result of such a misinterpretation as well, as illustrated in Fig. 26c. The literal ‘Rg’ present in the original source of the same structure, USPTO patent US6835754, is not present in the structure provided to PubChem in SID 143366847, being replaced with a carbon atom. Additional examples can be found for erroneously annotated rutherfordium (Rf) atom in CID 59869973, from SID 142371601, originating from USPTO patent US6214862 (Fig. 26d). There, fragments annotated as R^1^ and R^2^ result in methyl groups in the deposited structure (again, due to replacement by a carbon atom). This structure contains a further example of a polonium (Po) atom that is annotated as a radical by PubChem standardization. Going back to the original patent, the ‘Po’ abbreviation indicates a protected hydroxyl group, but lost when the structure is added to PubChem. Another case of erroneous polonium atoms is exemplified in Fig. 26e. The structure in CID 60160982 (from SID 144104720) is labeled as ‘polyacenaphthylene’ by its depositor, which is more accurately represented as shown in Fig. 26e (iii). Apparently, in this case, ‘Po’ was originally used to indicate a polymer. Other abbreviations resembling atomic symbols that are used as abbreviations for fragments are U (uranium, CID 60130730, SID 143372400, USPTO patent US20040242832, Fig. 26f), V (vanadium) and W (tungsten, CID 60104420, SID 143231020, patent US6770648, Fig. 26g). In Fig. 26g (iii), the ‘-X’ substituent that can be in 5- or 6-position in the ring as annotated is entirely lost in the deposited structure. Actinium (Ac) atoms can also be falsely annotated as shown in Fig. 26h (CID 60101345, SID 143213558, patent US6903102). There, the abbreviation ‘Ac’ was used to describe an acetyl group.

In order to estimate an upper bound for the number of atom types and atom environments possibly affected by the misperceptions outlined above, Table 3 provides counts for commonly mis-annotated elements. The total numbers of atom environments derived from the commonly mis-annotated elements for Substance are 679, 31,163, 157,164, and 298,715 for radius *r* = 0, 1, 2, and 3, respectively. For Compound, the numbers are considerably lower, with 144, 978, 3,652, and 6,980, for atom environments with radius *r* = 0, 1, 2, and 3, respectively.

**Table 3:** Statistics for elements potentially originating from misperceived abbreviations in PubChem Substance and Compound

Atomic symbol	Atom types		Atom environments					
	*r* = 0		*r* = 1		*r* = 2		*r* = 3	
	Sbst	Cmpd	Sbst	Cmpd	Sbst	Cmpd	Sbst	Cmpd
V	157	25	4,818	232	16,194	656	25,686	1,170
Rb	28	4	1,388	7	9,054	9	19,098	12
Y	80	10	7,432	24	57,213	47	1,16,554	78
Rh	102	21	3,133	71	9,231	122	14,561	167
W	25	31	6,280	439	28,382	2,462	47,976	5,116
Re	126	21	2,413	81	8,004	116	12,587	140
Po	13	6	76	58	182	154	247	214
Ra	12	3	1,527	7	11,355	9	24,073	9
Ac	18	1	395	–	3,919	–	15,461	–
U	101	21	2,744	59	9,419	77	14,791	74
Rf	17	1	957	–	4,211	–	7,681	–

Elements are ordered by atomic number. For each combination of repository and radius, the number of unique fragments containing the respective atom element is listed. Substance and Compound are abbreviated as ‘Sbst’ and ‘Cmpd’, respectively.

**Fig. 27:**
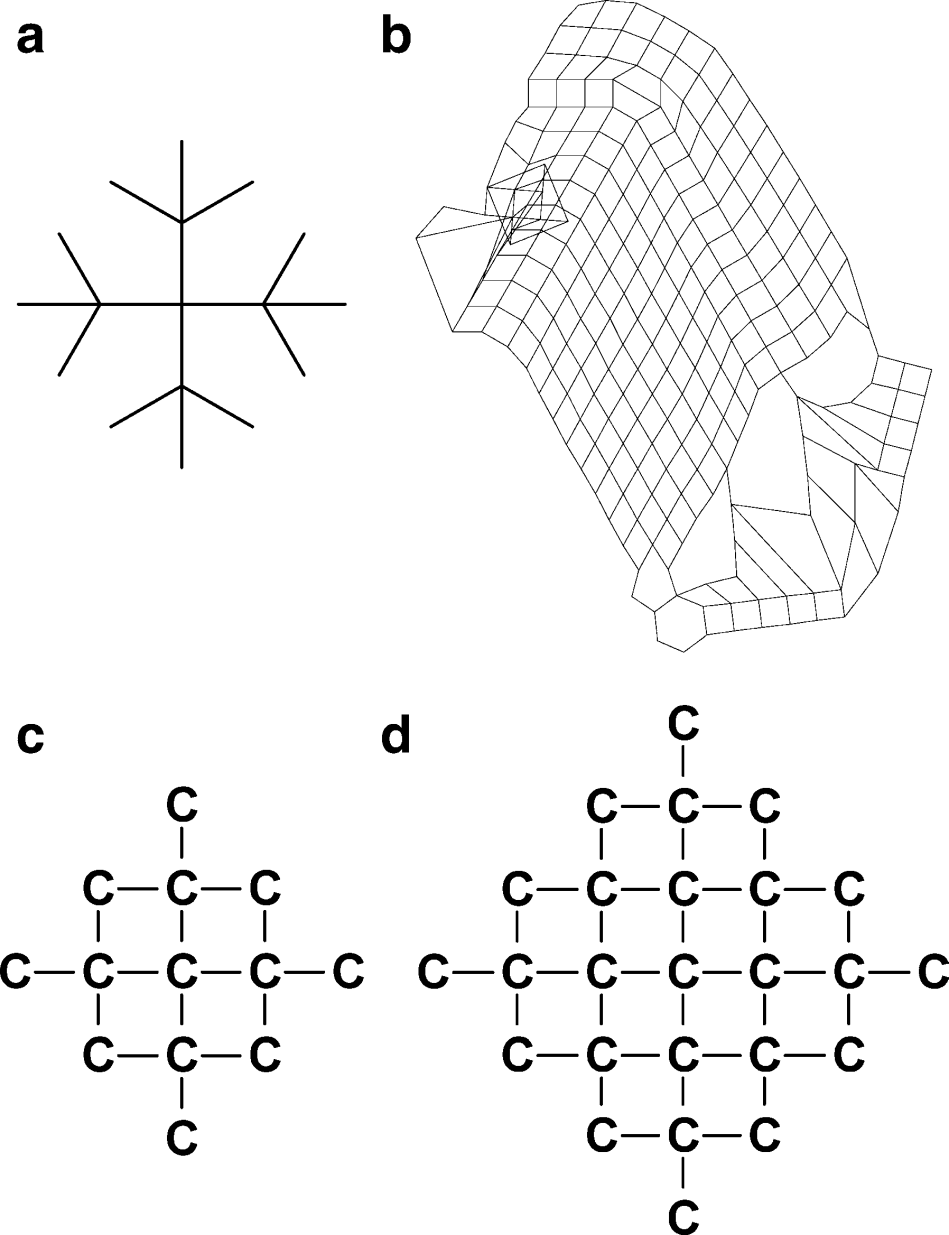
Implausible ‘valid’ structures in PubChem Compound. **a** Tetra-*tert*-butylmethane (CID 15123361), **b** CID 20695696, **c** Atom environment with radius *r* = 2 extracted from (**b**) used as filter for implausible structures (outgoing connections omitted for clarity), **d** atom environment with radius *r* = 3 extracted from (**b**) used as filter for implausible structures (outgoing connections omitted for clarity).

Even when considering only organic elements, erroneous structures consisting of valid atoms can be found in PubChem Substance and Compound, and will have an influence on atom environment diversity studies. Tetra-*tert*-butylmethane (Fig. 27a) is a hypothetical structure that has been identified as the smallest saturated and acyclic hydrocarbon that cannot be synthesized [73, 74]. Yet, it is present in Compound as CID 14123361 with the corresponding SIDs 137126462 and 143067637. Another example has been identified by Kolodzik et al. [75] as illustrated in Fig. 27b. The structure in CID 20695696 corresponds to SIDs 33924192 and 142503477. The latter references USPTO patent US20020016531 as source, but no such chemical structure is specified in the patent, rather a chemical drawing package was used to represent a grid in a patent figure. The presence of these structures in PubChem is clearly erroneous. The use of atom environments may be an effective way to identify such ‘valid’ but implausible structures. For example, an atom environment with radius *r* = 1 can describe tetra-*tert*-butylmethane, with a tetra-coordinated carbon with each adjacent atom being of the same type. Expanding such an atom environment to radius *r* = 2, as illustrated in Fig. 27c, one can identify 247 entries in Compound containing this pattern. Taking this one step further, expanding the atom environment to radius *r* = 3, as illustrated in Fig. 27d, yields 159 entries in Compound, a subset of those identified using the *r* = 2 environment. After visual inspection, the 247 identified compounds appear to be erroneous structures that do not represent ‘real’ chemicals. By selective manual examination of atom environments, one could generate filters to remove such classes of sterically implausible chemical structures.

These examples help to showcase that not all atom types and atom environments generated by this study resemble fragments of valid and real chemical structures. The belief is that these sorts of issues are rare and that they apply only to a relatively small number of atom types and atom environments. It also helps to demonstrate that simple atom environments (e.g., *r* = 0, *r* = 1, and beyond) are more tractable for manual curation than all of PubChem. Conceivably, these environments could be used as a ‘sanity check’ of real or plausible chemistry, and therefore are worthy of further investigation for chemical structure normalization and quality assurance (QA) purposes. The importance of this cannot be understated, as every new contribution to PubChem might exhibit structural elements previously unknown to the standardization protocols. A comparison of atom types and atom environments between Compound, Substance, and structures being contributed could automatically identify new representations and suggest structure examples for curation that can then be used for further refinement of standardization methods.

## Conclusions

The chemical structure contents of the PubChem Compound and Substance databases was examined as a function of atom types and atom environments. The relative novelty of chemical structure fragments found in PubChem is considerable. The percentage of atom environments located in only a single PubChem Compound record is 10.6, 28.5, 39.1, and 44.1% for atom environment *r* = 0 (atom type), 1, 2, and 3 (ECFP_6 like), respectively. Considering many chemical structures are synthesized for novelty purposes, this may not be completely surprising. Interestingly, the relative rate of increase of new atom environments, while still substantial, slows dramatically when examined as a function of increasing atom environment radius in PubChem Compound, with a 69, 42, and 6-fold increase for 0–>1, 1–>2, and 2–>3, respectively. This suggests that there is still considerable room for chemists to pursue novel chemical structures using only new combinations of smaller (e.g., *r* = 2) atom environment fragments.

Further emphasizing this point, plots of the incidence of atom environment fragments at various sizes show a log/log behavior. In some ways, this may suggest that chemists lack imagination in that the majority of chemical structures contain one or more of the same basic molecular fragments. One could also easily argue the opposite point, in that chemists are constantly pushing into new and unexplored areas of chemistry and are rarely using the same atom fragments twice. In the end, it seems very clear that chemists have plenty of room to explore new and sparsely explored chemistry space and, therefore, make many new discoveries for some time to come.

The analysis of PubChem Compound was compared to similar studies performed over 40 years ago by Crowe et al. and Adamson et al. of CASRS chemical structures. A near eightfold (8×) increase in *r* = 1 atom environments (‘augmented atoms’, atom and its nearest neighbors) was found. While this result can only be considered an upper bound, due to the use of structure sampling by the earlier studies and the relatively high rate of singletons found in the PubChem analysis, it does imply a substantial increase in the capability of chemists to synthesize and isolate novel chemistry as a function of time, with a noted increase in the prevalence and popularity of nitrogen and oxygen containing atom environments *now* as opposed to *then*. The supporting information provided in this study should allow for future comparisons on the progress and trends of chemists.

The differences between the PubChem Substance and Compound databases were examined, in part, by using examples of atom environments of increasing size unique to each repository. This study noted the count of unique atom environments in Substance is greater than it is in Compound. This is due to the fact that structures in Substance undergo structure standardization and have to pass validity filters before becoming part of Compound. This ‘sanity’ step dramatically reduces the count of atom environments by removing implausible chemistry (e.g., five bonds to carbon) and by normalizing varying functional group representations. These differences also help to emphasize the effect of PubChem standardization protocols for preferred atom types and particular tautomeric/resonance forms such that they could be used as the basis for a fragment-based structure normalization procedure.

The analysis of the Compound database is particular helpful to understand and characterize the diversity of molecular fragments found in known chemicals. Given the limited number of atom environments up to *r* = 3 (ECFP_6 like), it may be possible to do a more thorough examination of observed fragments to improve the efficiency of chemical information algorithms, such as those for chemical structure searching or virtual screening. Furthermore, the results of this study highlight that further refinement of standardization procedures in PubChem will be beneficial.

## Methods

In this analysis, the OpenEye Scientific, Inc. OEChem C++ toolkit was used for the representation of atoms, bonds, and molecules [57].

### Structure preprocessing

Most standard formats for structure representation in chemical information, such as SMILES [11, 12] and connection table file formats [23–25] do not require the specification of explicit hydrogen atoms in a chemical structure or implicit hydrogen atom counts. Instead, a standard valence model is employed, where implicit hydrogen atom counts are determined from (among other things) the atomic number, explicit atom valence and formal charge. Standard valence models can vary between file formats and software implementations. In PubChem Substance, the presence of explicit hydrogen atoms are nearly always limited to chemical structures with a hydrogen atom involved in the configuration of a stereocenter or to specify a particular isotope form. Consequently, most non-hydrogen atoms in Substance have non-saturated valences, and the chemical structures do not represent valid chemistry without additional processing to assign implicit hydrogen counts. In order to account for these effects, Substance records were subjected to a standard valence model prior to atom environment analysis by invoking the OEChem C++ toolkit [57] function OEAssignMDLHydrogens. PubChem Compound is derived from Substance through automated structure standardization protocols, including the adjustment of implicit hydrogen atom counts and subsequent assignment of explicit hydrogen atoms. For the purpose of this analysis, all explicit hydrogen atoms of substances and compounds were converted to implicit hydrogen atom counts using the OEChem C++ toolkit [57] function OESuppressHydrogens with all Boolean parameters set to ‘false’. Please note that this explicit-to-implicit hydrogen atom conversion removes all explicit hydrogen atoms, including those with specific hydrogen isotopes, affecting 98,342 deuterium and 21,039 tritium containing substances, as well as 56,725 deuterium and 8,909 tritium containing compounds, respectively.

### Atom types

In this study, we employed two atom typing schemes. For an adequate comparison of fragments in PubChem Compound to the results of an ‘augmented atom’ study of the CASRS published by Adamson et al. [47], atoms are characterized by their atomic number as sole feature. For a more detailed analysis of circular atom environments in PubChem Substance and Compound, atoms are characterized by six properties: (1) atomic number; (2) formal charge; (3) implicit hydrogen count; (4) explicit degree; (5) valence; and (6) participation in a conjugated (aromatic) system. The atom “explicit degree” is the number of explicitly connected atoms. The atom “valence” equals the sum of all incident sigma and pi bonds. The number of “incident sigma bonds” is described by the sum of “implicit hydrogen count” and “explicit degree”. The number of “incident pi bonds” is the sum of bond orders of explicitly connected atoms minus the “explicit degree”. This atom characterization approach allows a description of the molecular context of an atom (environment) without having to include the next layer of atoms as pseudo atoms as in other approaches [76]. Atom aromaticity was perceived using the OEChem C++ toolkit function OEAssignAromaticFlags in combination with the aromaticity model OEAroModelOpenEye. In the specific case of the comparison with CASRS, the OEAroModelMDL was used, as it allows for a more “apples to apples” comparison to the older study by allowing only six-membered rings of carbon and nitrogen to be aromatic, provided they satisfy the ‘Hückel 4n + 2′ rule [58, 59] (i.e., atoms are sp^2^-hybridized).

### Bond types

In this study, we employed two bond typing schemes. For an adequate comparison of fragments in PubChem Compound to the results of an ‘augmented atom’ study of the CASRS published by Adamson et al. [47], bonds are characterized by their covalent bond order (single, double, triple), and presence in ring or chain, plus an additional ‘aromatic’ ring bond type. Bond aromaticity was perceived using the OEChem C++ toolkit [57] function OEAssignAromaticFlags in combination with the aromaticity model OEAroModelMDL. For a more detailed analysis of circular atom environments in PubChem Substance and Compound, four different bond types are distinguished: single, double, triple, and aromatic. Bond aromaticity was perceived using the OEChem C++ toolkit [57] function OEAssignAromaticFlags in combination with the aromaticity model OEAroModelOpenEye. In addition to covalent bonds, PubChem defines and actively uses three non-standard bond types: ionic, complex and dative bonds. In this analysis, these non-standard bond types were completely ignored.

### Atom environments

**Fig. 28:**
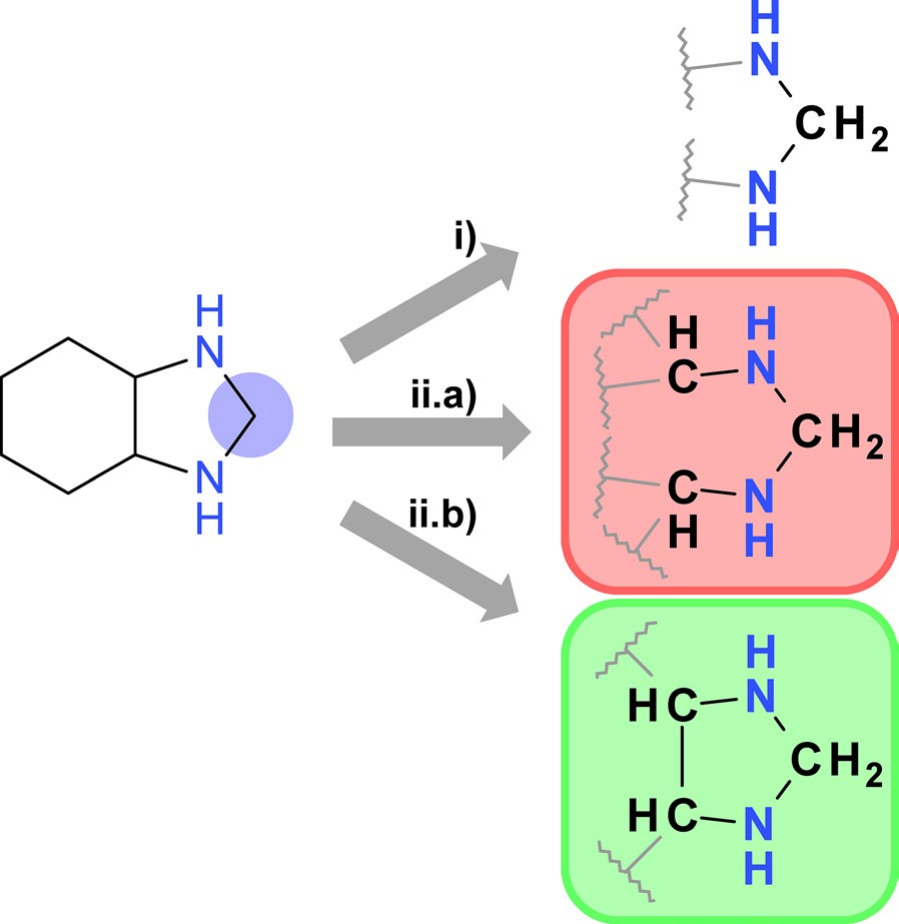
Bond inclusion in atom environment generation. Atom environments contain all bonds between included atoms. The effect is illustrated using the example of perhydrobenzimidazole (CID 21866348), the highlighted atom is the center atom in this example. *i* Atom environment with radius *r* = 1, *ii.a* Atom environment with radius *r* = 2 without including all connecting bonds, the five-membered ring remains open, *ii.b* atom environment with radius *r* = 2, including all connecting bonds closes the five-membered ring and enables distinguishing this case from branching scenarios.

**Fig. 29:**
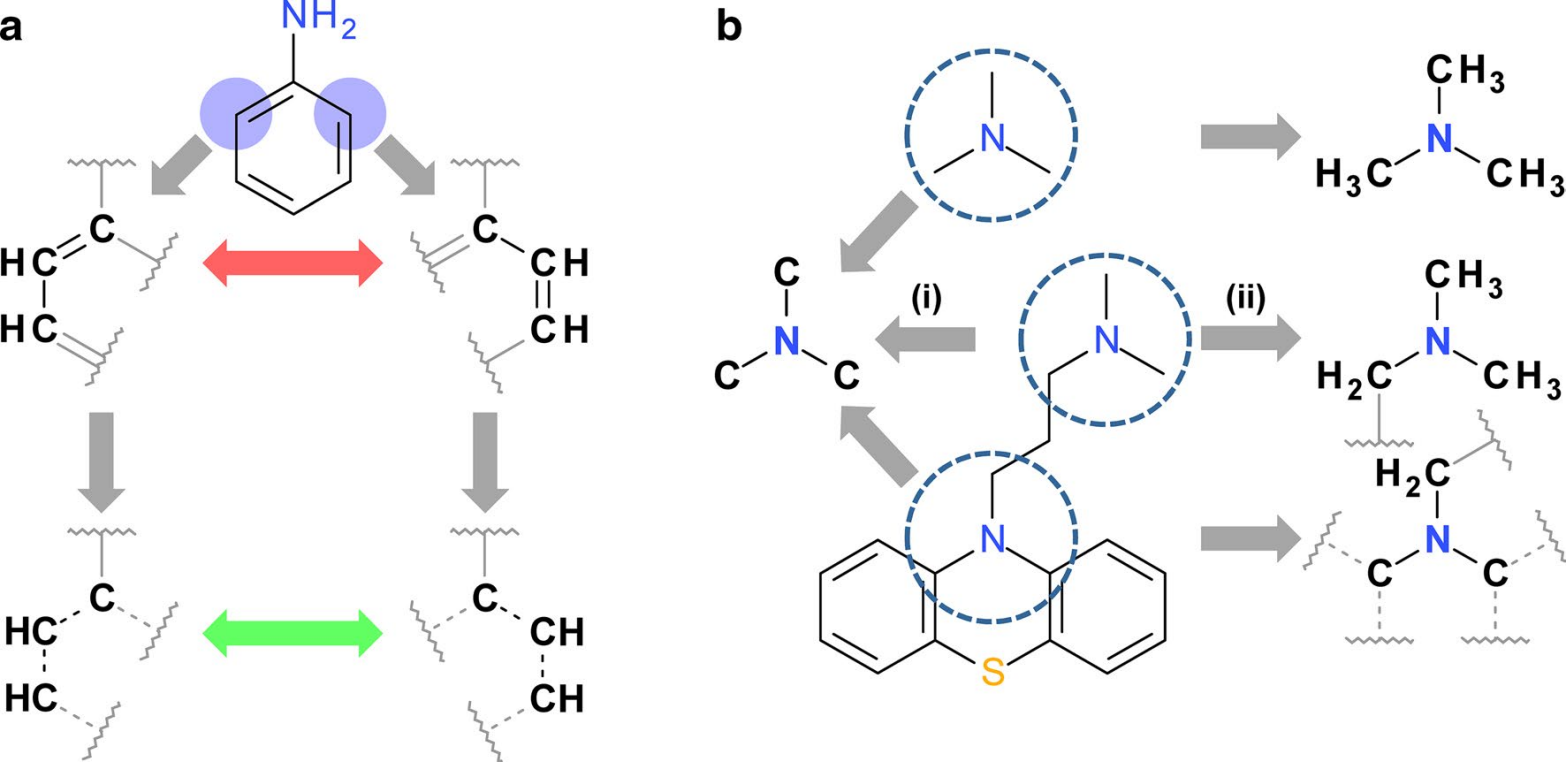
Aromaticity and connectivity in atom environment generation. **a** Influence of aromaticity perception annotation on atom and bond types using the example of aniline (CID 6115). If atom environments with radius *r* = 1 are generated around the highlighted atoms without aromaticity perception and annotation prior to atom environment generation, the resulting environments are not identical. Aromaticity perception and annotation and subsequent atom environment generation yields the intended result of two identical instances of the same environment. **b** Influence of encoding on connectivity information in atom types using the example of trimethylamine (CID 1146) and promazine (CID 4926). *Dashed circles* indicate atom environments of radius *r* = 1 that have identical element connectivity ‘N(C)(C)(C)’. **b** *i* If only the respective atomic number is considered as atom property, the three indicated atom environments cannot be distinguished. **b** *ii* Expanding the atom types to connectivity by including number of implicit hydrogen atom counts, explicit degree and valence allows to recognize all three fragments as being different*. Light gray* bonds for clarification of connectivity and valence.

Atom environments combine atom types and bond types into larger fragments. In this study, we employed the concept of circular atom environments centered on a particular atom referred to as the center atom. An atom environment of radius “*r*” contains all atoms in the molecule with a topological distance *r* or smaller to the center atom [55], and all bonds between them. The topological distance between two atoms is measured as the number of bonds on the shortest path between them. Consequently, atom environments with radius *r* = 0 include only the atom type of the center atom. Atom environments with *r* = 1 contain the center atom, all atoms adjacent to the center atom (nearest neighbors), and all the bonds between these atoms (those connecting the center atom with its neighbors and those between the neighbor atoms, if any exist). The inclusion of all bonds between atoms in an atom environment facilitates better separation between atom environments in ring-close scenarios as illustrated in Fig. 28. The advantages of including aromaticity and connectivity information in atom and bond types are illustrated in Fig. 29.

Atom environments with *r* > 0 were not generated with terminal atoms as center atoms, referring only to atoms that are adjacent to one other atom. These terminal atoms are included in the environment originating from the adjacent—non-terminal—partner. However, this exclusion of terminal atoms means that mono- and di-atomic structures are excluded from any atom environment analysis when *r* > 0, as they consist exclusively of terminal atoms. In Substance, this leads to 1,797 mono-atomic and 3,795 di-atomic structures being excluded from the atom environment *r* > 0 analyses. In Compound, this leads to 448 mono-atomic and 1,306 di-atomic structures being excluded from the atom environment *r* > 0 analyses. Statistics for these excluded structures are provided in the supporting information. (See Additional file 1: Figures S4, S5) Terminal atoms are included in the atom environment *r* = 0 (i.e., atom type) analysis.

In order to generate canonical representations for atom environments, we used functionalities from the OpenEye Scientific Software, Inc. OEChem C++ toolkit [57]. Atom environments were encoded as simplified molecular-input line-entry system arbitrary target specification (SMARTS) patterns [77]. For each atom in an OEMol object that represents a PubChem substance or compound record, a SMARTS representation was generated according to the encoding described in Tables 4 and 5. This string representation of atom properties was set as the atom name by invoking the method *SetName*, after setting the atomic number to ‘0’. Atom environments were programmatically generated as OEMol objects containing only the atoms of a molecule with a shortest topological distance to the center atom that is lesser or equal to the atom environment radius, and the bonds between those atoms. A canonical string representation of the OEMol object representing the atom environment was generated by invoking the function *OECreateSmiString* with the OESMILESFlag values ‘Canonical’ and ‘SuperAtoms’. As the atomic number was set to ‘0’, canonicalization uses the string value set as atom name for prioritization during the canonicalization procedure. These canonical representations of atom environments, as provided in Additional file 4, were the basis for our study.

**Table 4:** Atom and bond primitives for encoding of ‘augmented atoms’ in SMARTS

Feature	SMARTS encoding	Special case
Atom primitives		
Element	# <atomic number>	
Bond primitives		
Single bond	–	@ (!@) for ‘in ring’ (‘not in ring’)
Double bond	=	@ (!@) for ‘in ring’ (‘not in ring’)
Triple bond	#	@ (!@) for ‘in ring’ (‘not in ring’)
Aromatic bond	:	

Encoding specific to the environment analysis performed in comparison to the results published by Adamson et al. [47].

**Table 5:** Atom and bond primitives for encoding of atom types and atom environments in SMARTS

Feature	SMARTS encoding	Special case
Atom primitives		
Element	Atomic symbol	Lower case indicating aromaticity
Formal charge	±<integer>	Uncharged represented as +0
Implicit hydrogen count	h<integer>	
Explicit degree	D<integer>	
Valence	v<integer>	
Bond primitives		
Single bond	–	
Double bond	=	
Triple bond	#	

Aromatic bonds are implied between aromatic atoms unless explicitly specified otherwise.

### Incidence and occurrence

In this study, atom environment frequency is expressed in terms of incidence and occurrence. Incidence refers to the absolute count or percentage of (substance or compound) records that *contain* a particular fragment. Occurrence refers to the absolute count or percentage of *all* fragments across *all* structures. Therefore, per chemical structure record, occurrence considers *all* fragments, while incidence considers only the *unique* fragments.

### Dataset

This study uses PubChem as it existed on January 14, 2013 with maximum SID 160,655,685 and maximum CID 70,680,246. For both data sets, only PubChem records searchable (‘live’) at that point in time were processed. PubChem Substance records with ‘auto-generated’ structures were excluded. In ‘auto-generated’ cases, no actual structure is deposited, but a reference to a PubChem Compound record is derived using chemical names and may include chemical name conversion using various approaches, including the OpenEye Scientific Inc. Lexichem C++ toolkit [78]. Lastly, the chemical structure for a given substance had to be fully specified. Therefore, substances containing arbitrarily defined atoms (pseudo-atoms) were excluded from this analysis. By these criteria, atom environments (*r* = 0, 1, 2, 3) were determined for 104,669,789 Substance records. All 46,704,121 ‘live’ records in Compound were also processed.

All atom environments (*r* = 0, 1, 2, 3) found are provided as supporting information in Additional files 3 (r = 0; atom types) and 4 (r = 1, 2, 3; atom environments) as SMARTS patterns. Usage of atom and bond primitives for encoding of augmented atoms and PubChem atom environments are detailed in Tables 4 and 5, respectively. Provided in this format, fragments can be visualized using appropriate techniques [79, 80], or readily imported into various toolkits. All SMARTS patterns supplied as supporting information have been tested for their validity by successfully parsing them through the OEChem C++ toolkit [57] function OEParseSmarts.

### Records

Records may be referred to as SID (substance identifier) for PubChem Substance records and CID (compound identifier) for PubChem Compound records. Atom environments that occur in only a single PubChem record are referred to as singletons.

## Additional files

Additional file 1:
**Supplementary Figures and Tables.**
Additional file 2:
**PubChem Compound augmented atoms.** This file contains all ‘augmented atoms’ generated from PubChem Compound for the purpose of comparison to the fragmentation studies of Adamson et al. performed on CASRS in 1971 [46]. Each line describes an augmented atom as SMARTS string and its respective incidence, tab-separated. File is sorted by descending incidence. Additional file 3:
**PubChem atom types.** This file contains all atom types generated in this study for PubChem Substance and PubChem Compound in SMARTS format. Each SMARTS string is followed by a semicolon-separated incidence vector that specifies in how many records in which repository the particular atom type was found. Order of repositories is PubChem Substance followed by PubChem Compound. File is sorted by lexicographical order of SMARTS strings. Additional file 4:
**PubChem atom environments.** This file contains all atom environments generated in this study for PubChem Substance and PubChem Compound in SMARTS format. Each SMARTS string is followed by a semicolon-separated incidence vector that specifies in how many records in which repository and with what radius the respective fragment was generated. As each, PubChem Substance and Compound, were fragmented with three radii in total (*r* = 1,2,3), the vector contains six values. Association between position in the vector, PubChem Substance/Compound, and atom environment radius is as follows: position 1) Substance *r* = 1; position 2) Substance *r* = 2; position 3) Substance *r* = 3; position 4) Compound *r* = 1; position 5) Compound *r* = 2; position 6) Compound *r* = 3. File is sorted by lexicographical order of SMARTS strings.
