# Supplementary material for: PubChem atom environments
Source: J Cheminform. 2015 Aug 19;7:41. doi: 10.1186/s13321-015-0076-4 (PMC4540750; doi:10.1186/s13321-015-0076-4)
Supplement: Additional file 1: — Supplementary Figures and Tables. [file 13321_2015_76_MOESM1_ESM.pdf]

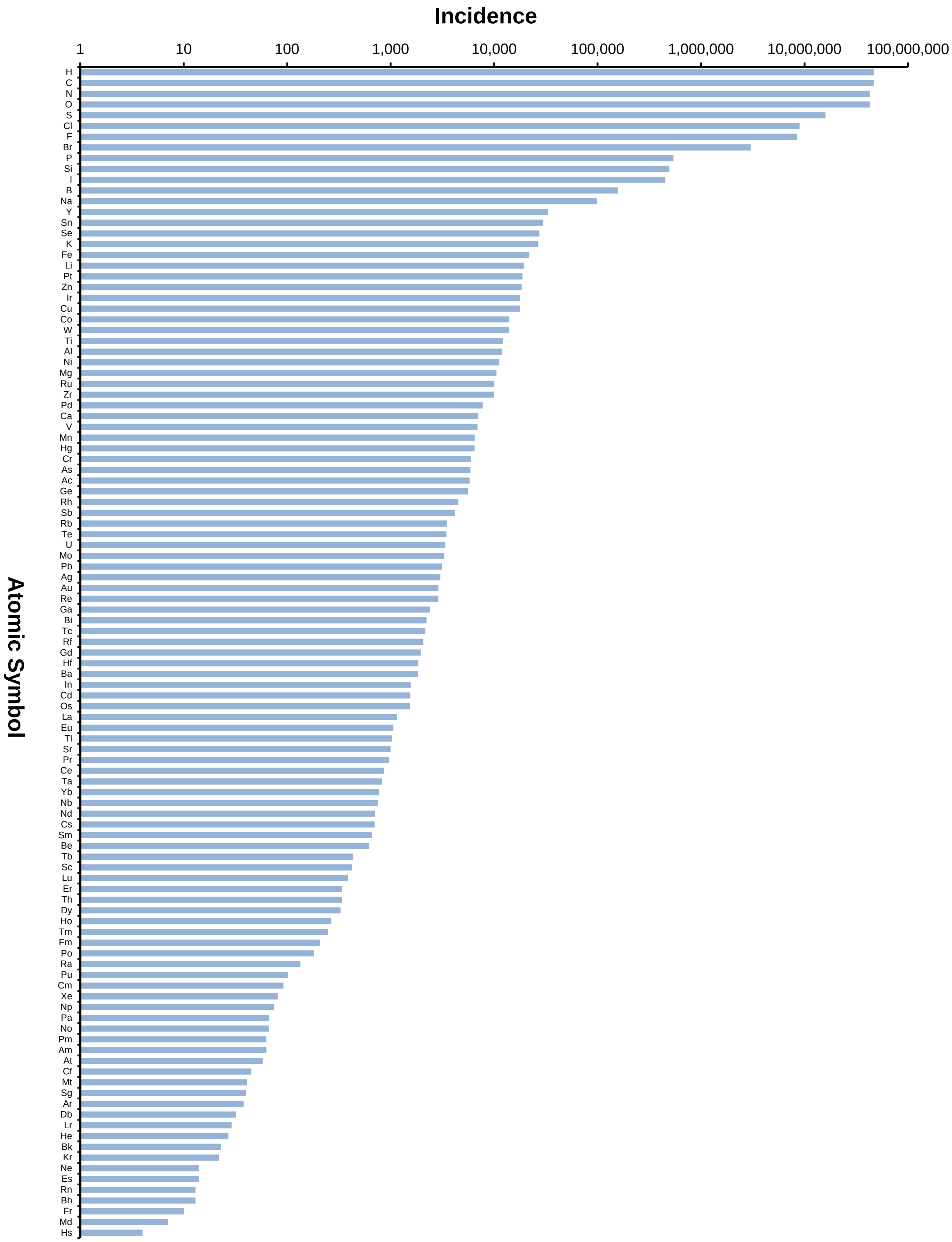

Figure S1. Rank/Frequency plot of element incidences in PubChem Compound.

Table S1. Elemental Analysis of PubChem Compound.

AN	Incidence	Occurrence	AN	Incidence	Occurrence	AN	Incidence	Occurrence
1	46,671,331	1,090,128,408	38	996	1,100	75	2,893	3,478
2	27	30	39	33,153	56,377	76	1,533	2,146
3	19,333	24,229	40	9,922	12,419	77	17,844	18,265
4	618	755	41	753	951	78	18,834	20,689
5	156,246	211,743	42	3,296	5,027	79	2,895	3,510
6	46,667,472	935,977,554	43	2,163	2,198	80	6,470	7,675
7	42,794,287	129,269,343	44	10,020	11,355	81	1,036	1,185
8	42,745,854	142,531,671	45	4,511	5,699	82	3,149	3,816
9	8,478,424	17,044,737	46	7,757	9,382	83	2,226	2,685
10	14	14	47	3,021	5,288	84	182	197
11	98,538	148,499	48	1,550	2,335	85	58	61
12	10,549	11,400	49	1,562	1,812	86	13	14
13	11,867	14,415	50	29,906	33,753	87	10	10
14	494,129	708,150	51	4,208	5,266	88	134	155
15	541,086	758,840	52	3,456	4,293	89	5,789	9,398
16	15,959,331	19,751,173	53	452,033	516,044	90	338	389
17	8,959,702	11,777,748	54	81	81	91	67	69
18	38	48	55	699	961	92	3,373	5,362
19	26,723	36,339	56	1,827	2,211	93	75	78
20	6,987	8,017	57	1,155	1,421	94	101	109
21	421	492	58	865	1,045	95	63	70
22	12,162	13,944	59	962	1,265	96	92	113
23	6,907	12,595	60	708	876	97	23	26
24	5,986	6,978	61	63	68	98	45	68
25	6,478	7,715	62	662	818	99	14	15
26	21,759	26,126	63	1,062	1,198	100	207	350
27	14,010	16,430	64	1,954	2,590	101	7	7
28	11,208	12,764	65	429	524	102	67	225
29	17,807	21,858	66	328	422	103	29	30
30	18,449	20,966	67	267	325	104	2,068	3,156
31	2,403	3,018	68	340	429	105	32	33
32	5,589	7,830	69	247	290	106	40	54
33	5,906	7,405	70	774	887	107	13	16
34	27,345	32,705	71	387	452	108	4	4
35	3,020,665	3,361,057	72	1,849	2,111	109	41	53
36	22	22	73	825	900			
37	3,490	4,381	74	13,971	30,317			

For every atomic number, the corresponding incidence and occurrence is provided. Data was used to generate Figure S1.

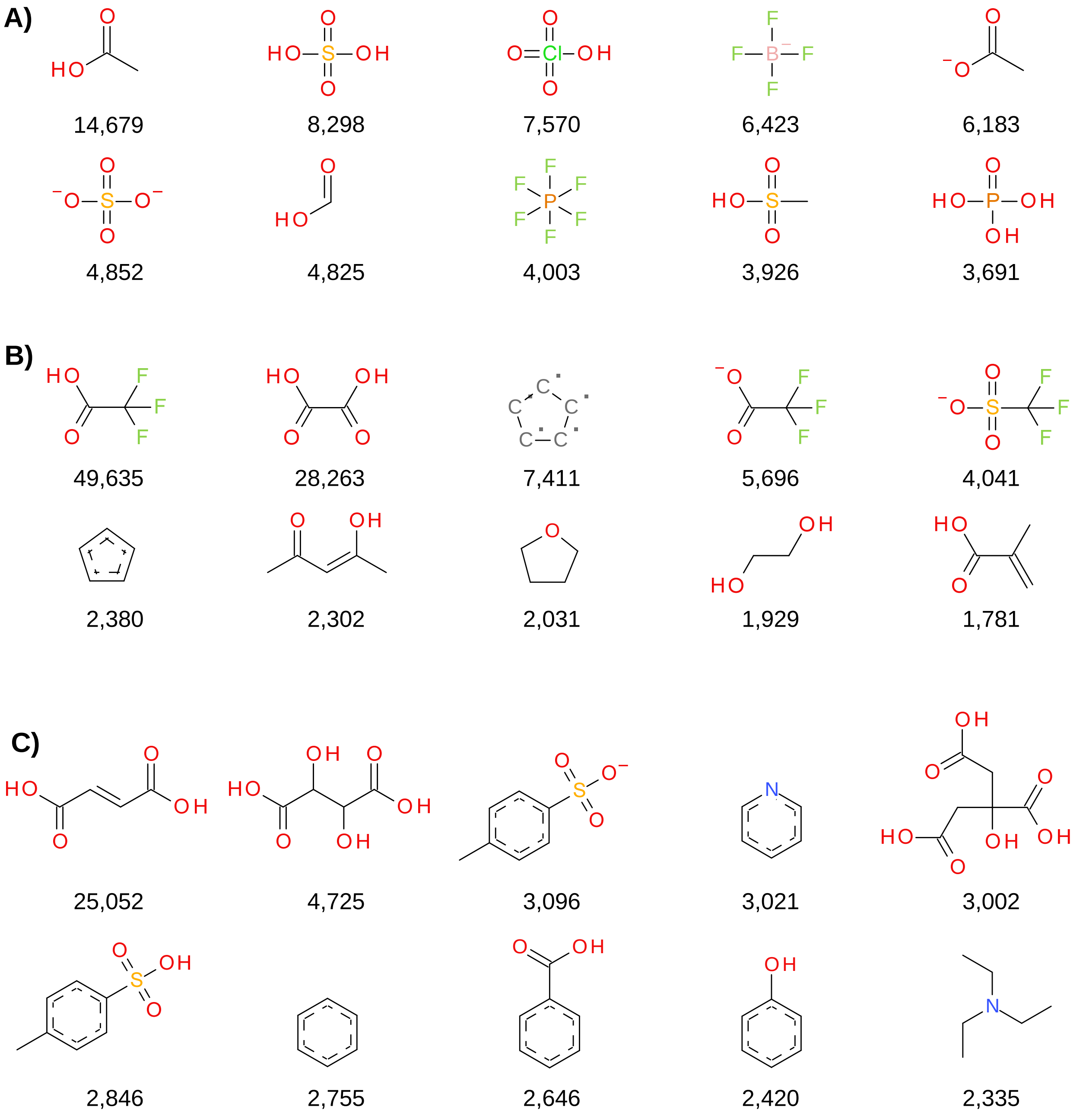

Figure S2. Top 10 most frequent structures fully described by atom environments in Substance. A) radius *r*=1; B) radius *r*=2; C) radius *r*=3. Environments are ranked by incidence, incidence is provided as absolute number. Dashed lines indicate aromatic bonds as perceived using the aromaticity model OEAroModelOpenEye in the OpenEye Scientific Software, Inc. OEChem C++ toolkit.

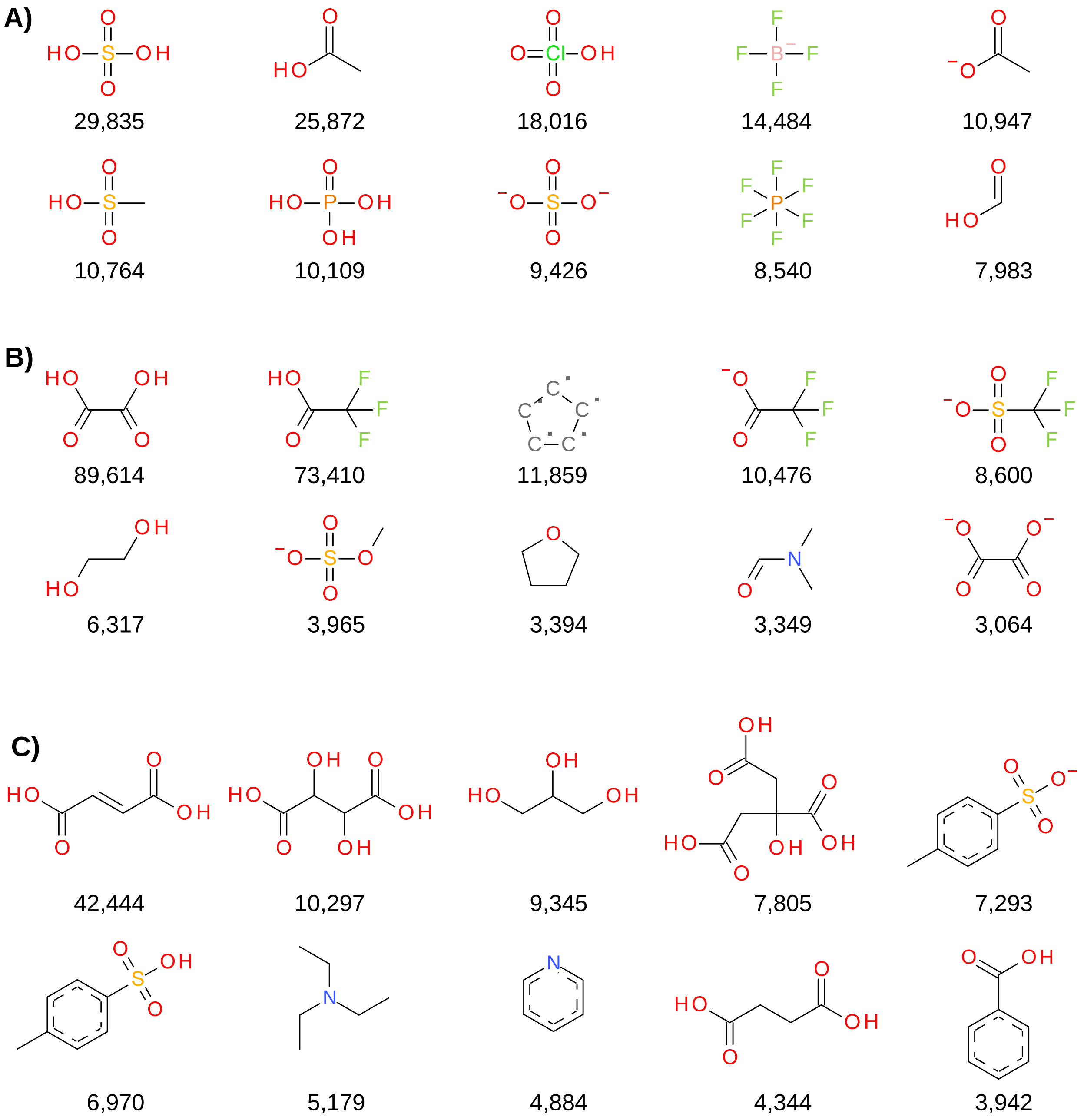

Figure S3. Top 10 most frequent structures fully described by atom environments in Substance. A) radius *r*=1; B) radius *r*=2; C) radius *r*=3. Environments are ranked by incidence, incidence is provided as absolute numbers. Dashed lines indicate aromatic bonds as perceived using the aromaticity model OEAroModelOpenEye in the OpenEye Scientific Software, Inc. OEChem C++ toolkit.

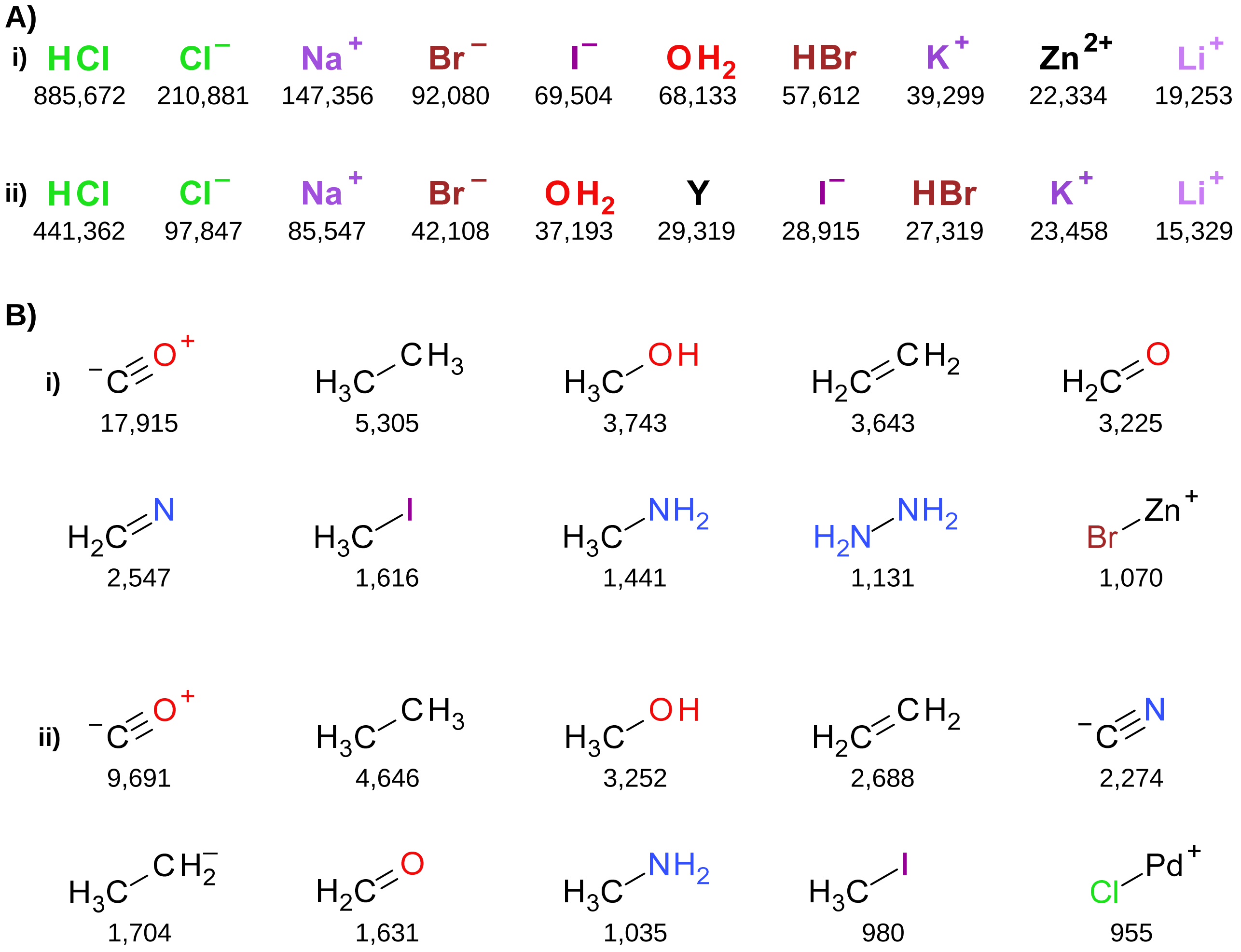

Figure S4. Top 10 most frequent unprocessed mono- and di-atomic structures. A) Mono-atomic structures; i) Substance; ii) Compound. B) Di-atomic structures; i) Substance; ii) Compound. Incidences as absolute numbers.

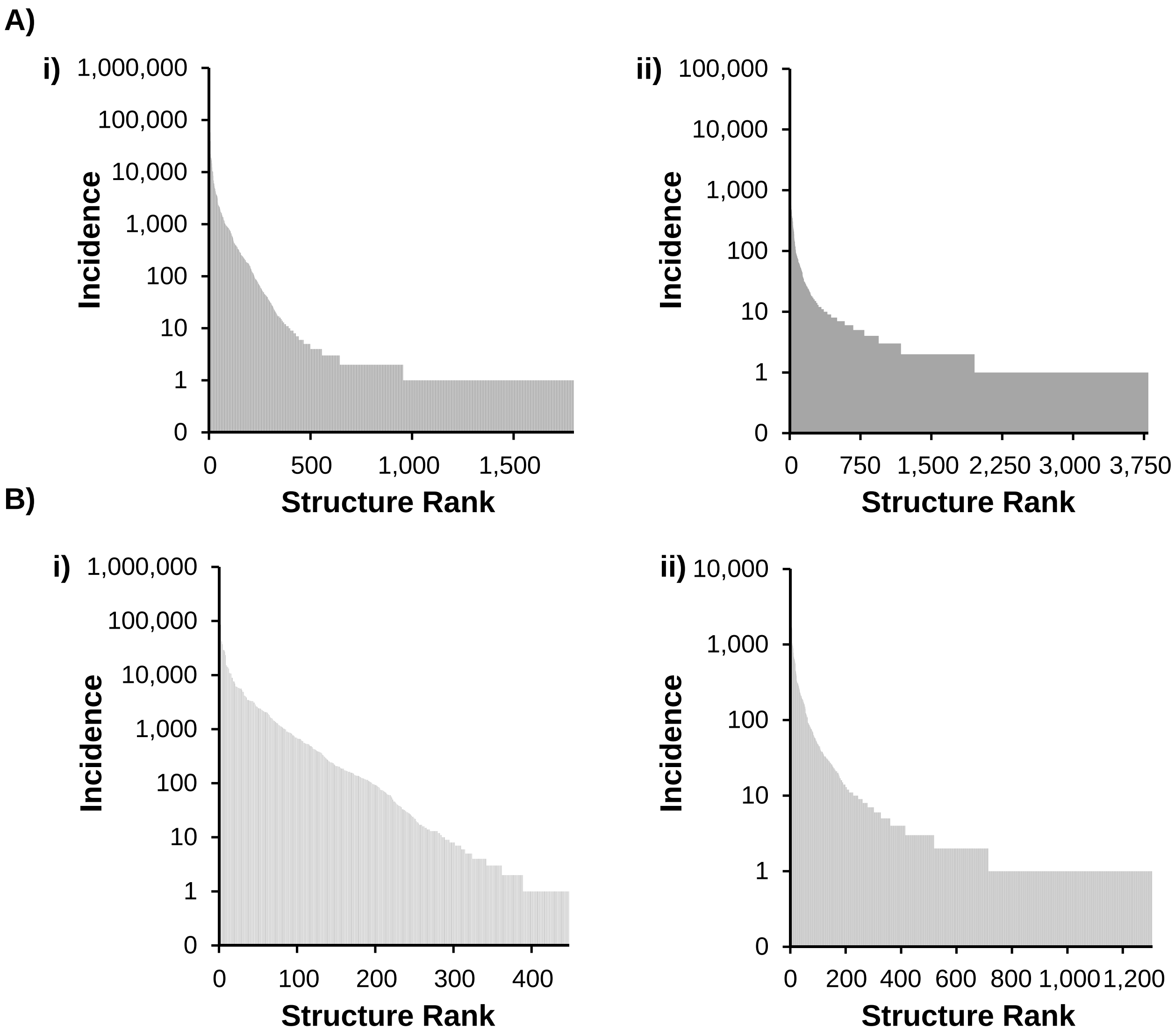

Figure S5. Rank/frequency plots of unprocessed mono- and di-atomic structures. A) Substance; i) monoatomic; ii) diatomic. B) Compound; i) monoatomic; ii) diatomic.
